# Differentially Regulated Host Proteins Associated with Chronic Rhinosinusitis Are Correlated with the Sinonasal Microbiome

**DOI:** 10.3389/fcimb.2017.00504

**Published:** 2017-12-06

**Authors:** Kristi Biswas, Brett Wagner Mackenzie, Sharon Waldvogel-Thurlow, Martin Middleditch, Mia Jullig, Melissa Zoing, Michael W. Taylor, Richard G. Douglas

**Affiliations:** ^1^Department of Surgery, School of Medicine, University of Auckland, Auckland, New Zealand; ^2^School of Biological Sciences, University of Auckland, Auckland, New Zealand; ^3^Auckland Science Analytical Services, University of Auckland, Auckland, New Zealand; ^4^Maurice Wilkins Centre for Molecular Biodiscovery, Auckland, New Zealand

**Keywords:** proteomics, bacterial 16S rRNA gene, inflammatory cells, biomarkers, PRRC2C, RAB14

## Abstract

The chronic inflammatory nature of chronic rhinosinusitis (CRS) makes it a morbid condition for individuals with the disease and one whose pathogenesis is poorly understood. To date, proteomic approaches have been applied successfully in a handful of CRS studies. In this study we use a multifaceted approach, including proteomics (iTRAQ labeling) and microbiome (bacterial 16S rRNA gene sequencing) analyses of middle meatus swabs, as well as immune cell analysis of the underlying tissue, to investigate the host-microbe interaction in individuals with CRS (*n* = 10) and healthy controls (*n* = 9). Of the total 606 proteins identified in this study, seven were significantly (*p* < 0.05) more abundant and 104 were significantly lower in the CRS cohort compared with healthy controls. The majority of detected proteins (82% of proteins identified) were not significantly correlated with disease status. Elevated levels of blood and immune cell proteins in the CRS cohort, together with significantly higher numbers of B-cells and macrophages in the underlying tissue, confirmed the inflammatory status of CRS individuals. Protein PRRC2C and Ras-related protein (RAB14) (two of the seven elevated proteins) showed the biggest fold difference between the healthy and CRS groups. Validation of the elevated levels of these two proteins in CRS samples was provided by immunohistochemistry. Members of the bacterial community in the two study cohorts were not associated with PRRC2C, however members of the genus *Moraxella* did correlate with RAB14 (*p* < 0.0001, rho = −0.95), which is a protein involved in the development of basement membrane. In addition, significant correlations between certain members of the CRS bacterial community and 33 lower abundant proteins in the CRS cohort were identified. Members of the genera *Streptococcus, Haemophilus* and *Veillonella* were strongly correlated with CRS and were significantly associated with a number of proteins with varying functions. The results from this study reveal a strong association between the host and microbes in the sinonasal cavity. Proteins identified as associated with CRS could be new targets for drug therapies and biomarkers for assessment of treatment efficacy.

## Introduction

Chronic rhinosinusitis (CRS) is defined by inflammation of the sinonasal cavities persisting for >3 months and occurs in 5% of the general population (Gliklich and Metson, 1995; Bhattacharyya, 2009; Fokkens et al., 2012). Symptoms associated with CRS include fatigue, headaches, nasal blockages and rhinorrhoea. The heterogeneous nature of this disease, all forms of which manifest with similar symptoms, and the complex combination of host genetics, immune response, and sinonasal microbiota has made it difficult for researchers to elucidate the pathophysiology of CRS (Hoggard et al., 2017a). One promising approach is to study CRS at the level of proteins (proteomics), which has the advantage of focusing on the phenotypic host response, rather than the underlying (and potentially variable) causative agents, gene expression levels or host genetics. Furthermore, proteomics is an ideal approach for studying complex diseases, such as CRS, in that this technique may identify potential biomarkers to help standardize the diagnosis of CRS subtypes and lead to more effective treatment for patients (Das et al., 2008).

The role of nasal mucus in sinus disease has been underestimated (Thornton and Sheehan, 2004; Rogers, 2007). As the first line of host defense from potential pathogens, mucus forms a physical barrier between the mucosa and the environment. Moreover, it is highly likely that proteins present within the mucus have specific protective functions that, when altered, could play an important role in the pathophysiology of CRS (Al Badaai et al., 2009). Investigations of cystic fibrosis (Sloane et al., 2005), obesity (Mardinoglu et al., 2014), and inflammation in the gut (Wasinger et al., 2016) have demonstrated that proteins which are differentially expressed between health and disease could play a role in disease pathophysiology. Similar approaches have also been applied to study allergic rhinitis and CRS (Tewfik et al., 2007; Al Badaai et al., 2009; Upton et al., 2011; Saieg et al., 2014; Tomazic et al., 2016). The majority of proteins found in these studies related to innate and acquired immunity, while only two of these studies were able to demonstrate significant differences in protein expression levels between CRS and healthy patients (Upton et al., 2011; Saieg et al., 2014). While informative, these initial studies are limited by low sample numbers, outdated technologies, and the targeting of specific proteins. Furthermore, it is increasingly apparent that the use of a multifaceted molecular approach can provide a better understanding of the inherent complexities of both the host-associated and microbial components of disease. This avenue of research could provide novel insights into the pathophysiology of CRS, and expose new potential drug targets.

The large body of recent work describing microbial community composition in patients with CRS has been inconsistent due to large variations observed among individuals (Feazel et al., 2012; Aurora et al., 2013; Biswas et al., 2015; Bordin et al., 2016; Cleland et al., 2016). One recent study was able to group CRS patients based on the relative abundances of members from the families *Pseudomonaceae, Streptococcaceae, Staphylococcaceae*, or *Corynebacteraceae* (Cope et al., 2017). These four microbially-based groups also had distinct gene expression patterns of host immune responses. However, the best supported hypothesis so far regarding the bacterial role in CRS pathogenesis is that CRS bacterial communities are dysbiotic or unbalanced (Wagner Mackenzie et al., 2017). The application of a multifaceted approach holds promise for expanding our current understanding of the microbe-host interactions in CRS, and chronic inflammatory disease more generally.

In this study, we used proteomics [specifically isobaric tag for relative and absolute quantitation (iTRAQ)] to identify proteins in the mucus collected from the middle meatus of 10 CRS and 9 healthy individuals. In addition, host immune cells (T-, B- cells, macrophages) within the biopsied middle meatus tissues were enumerated, and bacterial community composition of the surface mucosa in the middle meatus was investigated. Our primary aim was to correlate sinus mucus proteins with disease state, host inflammatory cell response and associated bacterial diversity.

## Materials and methods

### Patient recruitment

Ten adult patients with CRS and nine control subjects undergoing endoscopic sinus surgery for non-CRS related reasons were recruited for this study. Healthy control patients undergoing endoscopic sinus surgery for reasons other than sinus inflammation were chosen, as collection of samples was done under general anesthesia endoscopically from the middle meatus. CRS is the disease on which our research is focused. Thus, patients with CRS were recruited by Richard Douglas, an ORL surgeon, for the purposes of this study. Patients with co-morbidities such as cystic fibrosis, aspirin exacerbated respiratory disease, immunodeficiency, and individuals under the age of 18 years, were excluded. CRS patients with (*n* = 4) and without (*n* = 6) nasal polyps were included. Antibiotics and prednisone usage in patients prior to surgery was not an exclusion factor based on recent evidence that this has a negligible effect on bacterial community composition (Hoggard et al., 2017b). Demographic data, symptom severity scores, Lund-Mackay scores and medical history were recorded for each patient. This study was approved by the New Zealand Health and Disability Ethics Committee (NTX/08/12/126), and written informed consent was obtained from all participants.

### Sample collection

Tissue was chosen for IHC to enumerate immune cells. Mucus from the middle meatus was collected for proteomics and microbiome analysis as mucus is the first line of defense against invading pathogens. The middle meatus was chosen as the study site as the majority of the mucus drainage in the sinuses occurs at this location. Accordingly, sampling at this site provides a representative sample of the sinuses. Our previous study compared the microbiomes of different sites within the sinuses of an individual and found little variability (Biswas et al., 2015). Thus, the middle meatus is a commonly used study site for research studies. Surface mucus samples for proteomic analysis were collected under general anesthesia from the middle meatus with endoscopic guidance prior to any surgical intervention using a sterile cotton swab rotated four times. In addition, paired sterile rayon-tipped swabs (Copan Diagnostics Inc., USA) from the left and right nostrils were collected for microbiological assessment. All swabs were placed in a sterile 2 mL tube and transferred to the laboratory on ice within 2 h, where they were stored at −20°C until further analysis. Biopsy samples from the middle meatus, collected during surgery, were placed into Carnoy's fixative (60% ethanol, 30% chloroform, 10% glacial acetic acid) and transported to the laboratory for host immune cell counts.

### iTRAQ labeling proteomics approach

Cotton swab samples were thawed and re-suspended in 200 μL of sterile saline water. A gentle vortex for 10 s was used to re-suspend mucus from the swabs. Based on pilot study results (data not shown), a protein depletion column was used to deplete the two most abundant proteins (IgG and serum albumin), to gain a better resolution of less abundant proteins. Re-suspended raw mucus samples (50 μL) were then put through a ProteoPrep IgG and serum albumin depletion column kit (Sigma-Aldrich, New Zealand), as per the manufacturer's instructions. Samples were standardized to 0.5 mg/mL of protein and submitted for iTRAQ labeling and liquid chromatography tandem-mass spectrometry (LC-MS/MS) analysis at Auckland Science Analytical Services, School of Biological Sciences, University of Auckland, New Zealand. The sample preparation for LC-MS/MS methods and proteomic data analysis is described in the Supplementary Material. In brief, on three separate runs, a fractionated pool of eight randomized samples was injected into a TripleTOF 6600 Quadrupole-Time-of-Flight mass spectrometer (Sciex). The resulting data were analyzed using bioinformatics as described previously (Xu et al., 2015).

Proteins present in at least three samples within each sub-group (A) CRSwNP vs. CRSsNP; (B) CRSwNP vs. healthy controls; (C) CRSsNP vs. healthy controls, were selected for further comparisons. Analysis was performed as described above and *p*-values were calculated based on two-tailed *t*-tests.

### Immunohistochemistry (IHC)

Biopsied middle meatus specimens were fixed in Carnoy's solution, embedded in paraffin and sections cut at 4 μm thickness. Sections for immune cell markers were stained with mouse monoclonal antibodies CD3, CD20, or CD68 (Leica Biosystems, Newcastle Upon Tyne, UK) at 1:500, 1:100, 1:50 dilutions respectively, following antigen retrieval in citrate buffer (pH 6) using a 2100 Retriever. Sections for the proteins were stained either with rabbit polyclonal PRRC2C (Novus Biologicals, Littleton, CO, USA) 1:25, or with rabbit polyclonal RAB14 (Abcam, Cambridge, UK) 1:150, following antigen retrieval in EDTA (pH 9) using a 2100 Retriever. The Novalink^TM^ Polymer Detection System (Leica Biosystems, Newcastle Upon Tyne, UK) was used according to the manufacturer's instructions. Human tonsil was used as a positive control for immune cell markers, human colon for PRRC2C and human kidney for RAB14. Slides were examined using a Leica DMR upright microscope and photographed with a Nikon Digital Sight cooled color camera (Nikon Corporation, Tokyo, Japan) using Nikon NIS Elements for image acquisition (Nikon Corporation, Tokyo, Japan). Cell counts for immune cells were conducted in Image J (NIH, Bethesda, MD) by two individuals independently and the results then combined.

### Bacterial community composition

Microbial cells from pairs of rayon-tipped endoscopically guided swabs were lysed using lysing matrix E bead-beating tubes (MP Biomedicals, New Zealand) and a Tissue Lyser (Qiagen®) as previously described (Biswas et al., 2015). The AllPrep DNA/RNA isolation kit (Qiagen®) was used as per the manufacturer's instructions to extract genomic DNA, which was eluted in 30 μL of sterile water. A negative extraction control was run simultaneously using 200 μL of sterile water.

The V3-V4 region of the bacterial 16S rRNA gene was amplified using primers 341F and 806R (Klindworth et al., 2013) as previously described (Hoggard et al., 2017b). In brief, PCR amplification reactions were run in duplicate containing ~100 ng/ PCR reaction of genomic DNA for 35 cycles. Negative extractions were also subjected to PCR amplification but had no detectable product. PCR products were combined and purified using Agencourt AMPure magnetic beads (Beckman Coulter Inc., USA) as per the manufacturer's instructions. Purified products were quantified and quality checked using a Qubit dsDNA High-Sensitivity kit (Life Technologies, New Zealand) and Bioanalyser High-Sensitivity DNA chips (Agilent Technologies, USA), respectively. Sample concentration was standardized and samples submitted to the sequencing provider (New Zealand Genomics Limited) for library preparation and sequencing by Illumina MiSeq. Raw sequences have been uploaded to SRA-NCBI database (BioProject ID: PRJNA390854).

### Data analysis

Sequences were quality filtered, merged, and arranged into operational taxonomic units (OTUs) based on 97% sequence similarity, using USEARCH (Edgar, 2010) as previously described (Hoggard et al., 2017b). Taxonomic assignment of OTUs against the SILVA 16S rRNA gene database (version 111) was performed in QIIME (version 1.8) (Caporaso et al., 2010). Alpha and beta diversity metrics (including UniFrac distances) were calculated in QIIME and visualized in Calypso http://cgenome.net/wiki/index.php/Calypso (Zakrzewski et al., 2016).

Spearman's correlations were calculated on the bacterial community and iTRAQ signal (protein abundance) datasets, then visualized in a heatmap using GraphPad Prism^TM^ software (version 7.03). Clustering of correlation scores was based on Bray-Curtis dissimilarity distance matrices, using R (version 3.2.5.). Non-metric multidimensional scaling (nMDS) based on a weighted UniFrac distance matrix was used to construct an ordination plot for the bacterial samples. ADONIS, a feature of the vegan package in R (R Core Team, 2014), was used to test for differences between the bacterial composition of CRS and healthy control groups. Linear discriminant analysis effect size (LDA-LEfSe) (Segata et al., 2011) was used to identify potential bacterial biomarkers from 16S rRNA gene datasets. LDA-LEfSe analysis was performed using a non-parametric Kruskal-Wallis sum-rank test with an alpha value ≤0.05, followed by the unpaired Wilcoxon rank-sum with an alpha score ≤0.05 and a one-against-all strategy for multi-class analysis. Those OTUs with an LDA log score above 3.5 were plotted.

## Results

### Study cohort characteristics

The measured patient variables in this study included age, ethnicity, gender, smoking status, asthma, revision surgery, and preoperative antibiotics/corticosteroids within the 4 weeks prior to surgery. There were no significant differences between the two cohorts (CRS and healthy controls) for any of the measured variables (Table 1). Nasal polyposis was observed in 40% (4/10) of the CRS patients.

**Table 1:** Patient information.

	**Healthy (*n*^#^ = 9)**	**CRS (*n* = 10)**	**Unadjusted test *p*-value**
Age^a^	47 (22–74)	52 (25–65)	0.724
European^b^	6/9 (66.6%)	9/10 (90%)	0.3034
Gender (female)^b^	3/9 (33.3%)	5/10 (50%)	1
Smoker^b^	1/9 (11.1%)	1/10 (10%)	1
Polyposis^b^	N/A	4/10 (40%)	0.0867
Asthma^b^	2/9 (22.2%)	5/10 (50%)	0.3498
Preoperative antibiotics^b^	0/9 (0%)	2/10 (20%)	0.4737
Preoperative corticosteroids^b^	0/9 (0%)	1/10 (10%)	1
Revision surgery^b^	0/9 (0%)	2/10 (20%)	0.4737
Total symptom score	N/A	14 (9–21)	
Lund-Mackay score	N/A	12 (9–24)	

a *Mann-Whitney test was used for continuous variables (p < 0.05)*.

b *Fisher's exact test was used for categorical variables*.

*N/A not applicable*.

# *n, number of individuals in each cohort*.

### Proteomics

A total of 606 proteins were identified, of which 111 (18%) were significantly different in abundance between the groups (*p* < 0.05). The overwhelming majority of these (104 proteins) were significantly less abundant in the CRS group, with only seven proteins significantly higher in CRS samples (Table 2). The average abundance of these significantly different proteins in healthy and CRS cohorts accounted for 23 and 24% of total protein signal, respectively. This suggests that the majority of detected proteins (495 out of 606 identified proteins) are not relevant to disease status and these accounted for a total average abundance of ~75%. Proteins were also put through a BLAST search to look for possible hits with putative CRS pathogens *Staphylococcus aureus* or members of the genera *Corynebacterium* and *Haemophilus*, but no matches were found.

**Table 2:** List of the seven significantly higher abundant proteins in the CRS cohort, compared with healthy controls.

**Significantly elevated proteins in CRS**	**Origin**	**Sub-cellular**	**H *n* = 9 (^#^avg signal)**	**CRS *n* = 10 (avg signal)**	***P*-value (two-tailed *t*-test)**	**% of total protein signal in H**	**% of total protein signal in CRS**
Angiotensinogen	Blood	Secreted	6 (−1.87)	7 (0.271)	0.046	0.0339	0.081
Ras-related protein Rab-14 (Fragment)	Others	Others	3 (−3.47)	4 (−0.34)	0.0113	0.0001	0.004
40S ribosomal protein S12	Others	Cytoplasm	3 (−2.81)	4 (0.734)	0.002	0.135	4.399
Protein S100-A9	Immune cells	Cytoplasm	9 (−0.501)	10 (0.277)	0.033	1.998	5.725
Cell division control protein 42 homolog	Others	Others	3 (0.328)	4 (0.194)	0.041	0.023	0.041
Low affinity immunoglobulin gamma Fc region receptor III-B	Immune cells	Membrane	6 (−2.291)	7 (0.428)	0.005	0.005	0.084
Isoform 3 of Protein PRRC2C	Others	Membrane	3 (−5.705)	4 (3.96)	0.00008	0.0001	4.173
Total relative abundance of elevated proteins in CRS							14.5
Total relative abundance of reduced proteins in CRS							9.539
Total relative abundance of elevated and reduced proteins in CRS							24.039

# *Average signal for a protein is calculated as the sample signal minus the internal reference signal, which is then averaged across samples (n = 9 for healthy and n = 10 for CRS)*.

*H- healthy; CRS- chronic rhinosinusitis*.

#### Origin of proteins

**Figure 1:**
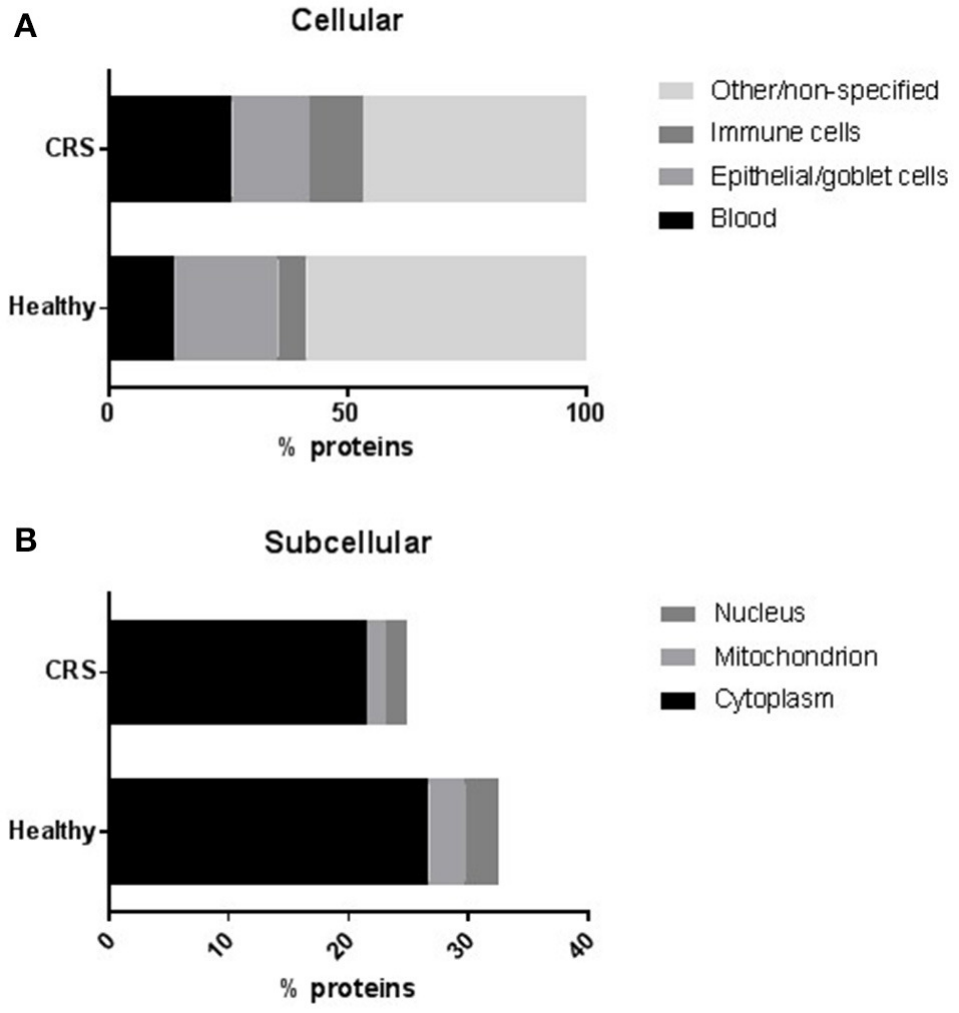
The distribution of proteins at **(A)** cellular and **(B)** subcellular levels. The number of proteins within each group were averaged and represented as a percentage of total proteins. Proteins that could not be assigned to the chosen categories or were non-specified on the protein databases were placed into a separate “other/non-specified” group in **(A)**. Proteins originating from blood or epithelial/goblet cells were differentially regulated (*p* < 0.05, *t*-test) in the CRS and healthy cohorts. At a subcellular level **(B)**, significantly fewer proteins originated from the nucleus and mitochondrion of CRS patients compared with healthy controls.

The 606 identified proteins were placed into four categories related to their origin: blood, epithelial/goblet cells, immune cells and other (non-specified). The CRS group contained significantly (*p* = 0.03) more proteins of blood, but not immune cell, origin compared with healthy controls (Figure 1A). The number of proteins originating from epithelium and goblet cells was significantly lower in the CRS group (*p* = 0.0008). The remainder of the proteins could not be described and these were placed into the other/non-specified category. Protein origin at the subcellular level was also investigated (Figure 1B), and revealed significantly fewer nuclear (*p* = 0.02) and mitochondrial (*p* = 0.0005) proteins in the CRS group. Cytoplasmic proteins were detected in high numbers and did not differ significantly between the two groups (26.7% CRS and 21.5% healthy).

#### Elevated proteins in CRS

**Figure 2:**
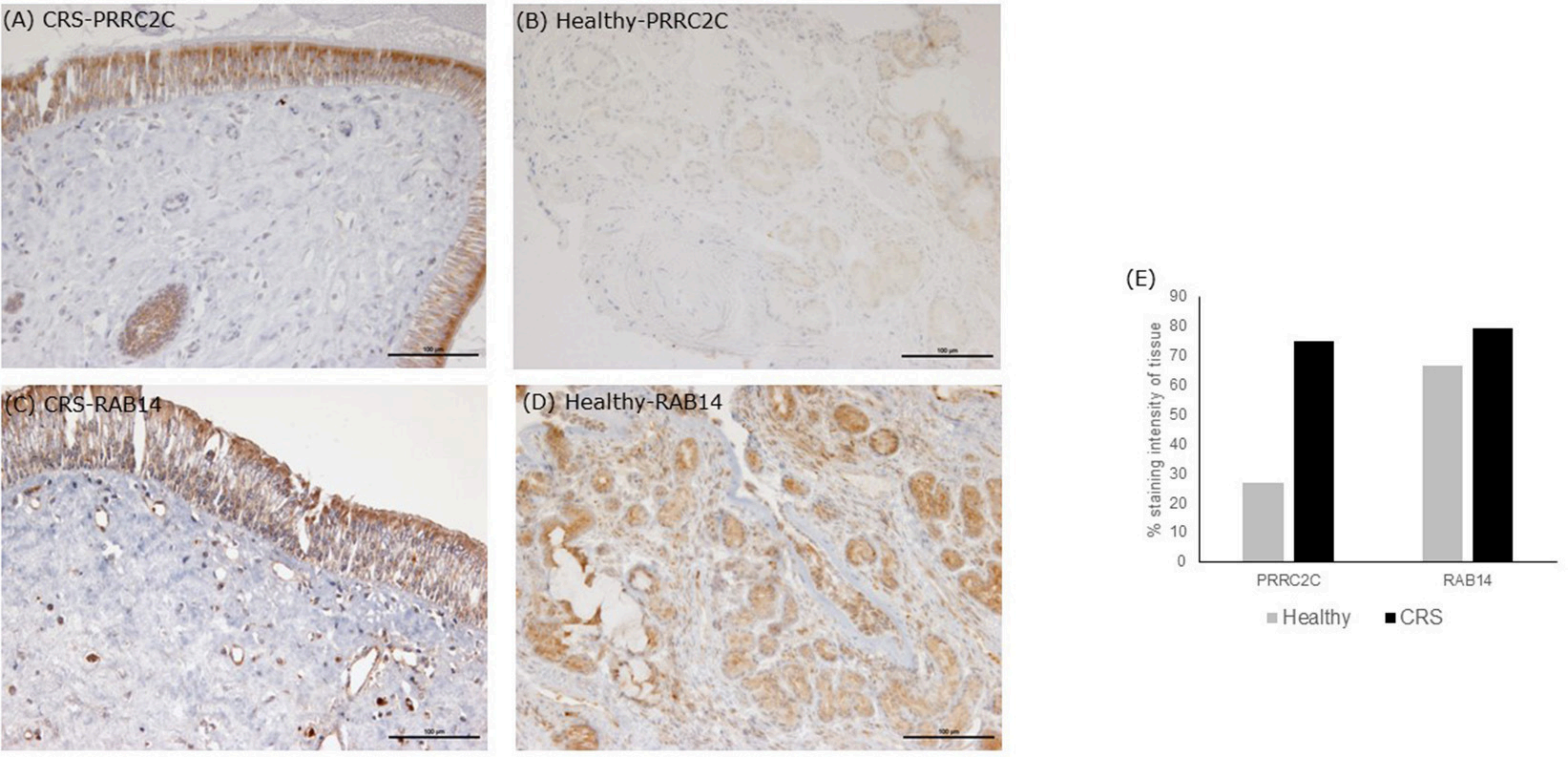
Immunohistochemistry staining of tissue sections. Tissue stains were compared with positive controls on the The Human Protein Atlas. **(A,B)** are representative images of middle meatus tissue biopsies from patients with CRS and healthy controls, respectively, stained with an antibody targeting protein PRRC2C. The protein PRRC2C was highly abundant in the epithelial cells, evident from the dark brown staining for CRS patients. **(C,D)** are representative of middle meatus tissue biopsies from patients with CRS and healthy controls, respectively, stained with an antibody targeting protein RAB14. RAB14 staining was observed within epithelial cells and macrophages of CRS patients with lighter staining within glands in healthy controls. Staining intensity is suggestive of protein abundance. Negative controls with no antibody did not show any staining. Grading stain intensity scores **(E)** were averaged across the samples for each cohort and shown as a percentage in the bar chart.

The seven significantly higher proteins in the CRS cohort accounted for 14.5% of total average iTRAQ signal (Table 2). Occurrence of these proteins within individuals in the cohort varied, as shown in Table 2. Isoform three of protein PRRC2C, involved in hematopoietic progenitor cell differentiation, was one of the seven elevated proteins observed in the CRS cohort. Notably, this protein was detected at >15,000-fold higher levels in CRS samples and was almost absent in healthy controls. The other distinctively elevated protein in CRS was Ras-related protein RAB14 (>22-fold higher). RAB14 is a membrane protein involved in trafficking cellular substances from the Golgi to the plasma membrane. The other five proteins of interest included angiotensinogen (origin from blood), 40S ribosomal protein S12 (others), protein S100-A9 (immune cells), cell division control protein 42 homolog (others), and low affinity immunoglobulin gamma Fc receptor Ill-B (immune cells). The two proteins with the highest difference were validated in the tissue samples using IHC (Figure 2). Staining of PRRC2C protein was observed on the surface of the epithelial cells. The intensity of the staining was subjectively measured. The greater the intensity, the more abundant the protein in the tissue. The staining of the tissue was compared with images of the nasopharynx in The Human Protein Atlas (www.Proteinatlas.org). RAB14 staining was observed within suspected macrophages and epithelial cells in CRS patients. Healthy controls displayed lighter staining within epithelial cells of glands. These images were also compared with positive controls on the THPA database. Negative controls, i.e., staining of tissue without any antibody, did not show any staining.

#### Lower abundant proteins in CRS

The 104 significantly lower abundant proteins in the CRS cohort were largely from the “others/non-specified” category, with a smaller percentage from epithelial/goblet cells. Some of these proteins included tight junction proteins (TJP2), ezrin (EZR), granulins, and prothymosin (PTMA) (Supplementary Table 1).

#### Sub-groups of CRS

Differences between CRSwNP and CRSsNP were also investigated. The abundances of four proteins differed significantly between CRSwNP (*n* = 4) and CRSsNP (*n* = 6) patients. These included three elevated proteins (calreticulin, alcohol dehydrogenase class 4 mu/sigma chain and peptidyl-prolyl cis-trans isomerase B) and one protein (peptidyl-prolyl cis-trans isomerase A) that was less abundant in the CRSwNP cohort.

Further comparisons were made between each CRS sub-group (CRSwNP and CRSsNP) and the healthy controls (Supplementary Table 2). A total of 19 proteins were significantly different in abundance between CRSwNP when compared with healthy controls. Similarly, 28 proteins were significantly different between CRSsNP and the healthy control cohort. The protein peptidyl-prolyl cis-trans isomerase A, which was significantly lower in CRSwNP vs. CRSsNP patients, was also significantly lower in CRSwNP compared with healthy control individuals. In addition, the abundance of protein alcohol dehydrogenase class 4 mu/sigma chain was significantly lower in the CRSsNP group compared to CRSwNP and healthy controls. These two proteins are of special interest and should be investigated as potential biomarkers to differentiate between the two sub-groups of CRS and healthy controls.

### Immunology data

**Figure 3:**
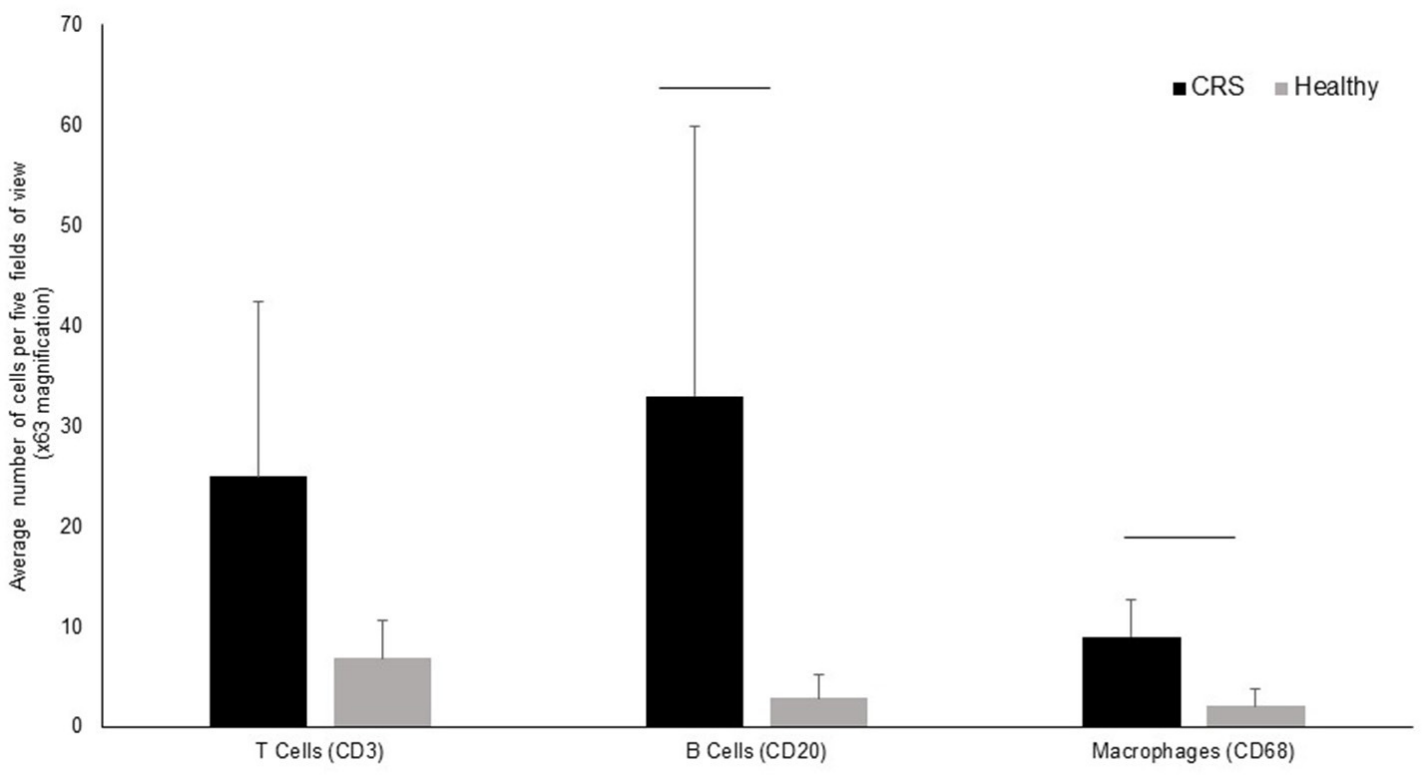
Enumeration of immune cells in middle meatus tissue of CRS (*n* = 9) and healthy (*n* = 5) individuals. The average number of T-cells, B-cells and macrophages are represented in the bar graphs along with standard deviation. Although cells were elevated in all three cell types, significant differences (*p* < 0.05 represented by a horizontal bar) between CRS and healthy cohorts was only observed for B-cells and macrophages.

T-cells, B-cells and macrophages were stained and counted in tissue biopsies of five healthy controls and 9 CRS patients, with biopsies unavailable for the remaining patients. Grouped results are shown in Figure 3, with data for individual patients shown in Supplementary Figure 1. All three cell types were elevated in CRS samples compared with controls, although only B-cells and macrophages were significantly different (*p* < 0.05) between the two groups.

### Bacterial community and correlation to protein abundance

A total of 111,033 high-quality 16S rRNA gene sequences, representing 475 unique 97%-OTUs, were recovered from the dataset. The average length of sequences was 437 nucleotides (min = 231 and max = 477), and samples were rarefied to 6118 sequences. There were no significant differences in alpha diversity when patients were grouped based on smoking status, asthma, previous surgery or disease status. The only exception was the Chao1 metric, an estimator of OTU richness, which was significantly higher in CRSsNP patients compared with CRSwNP and healthy controls (*p* < 0.05).

**Figure 4:**
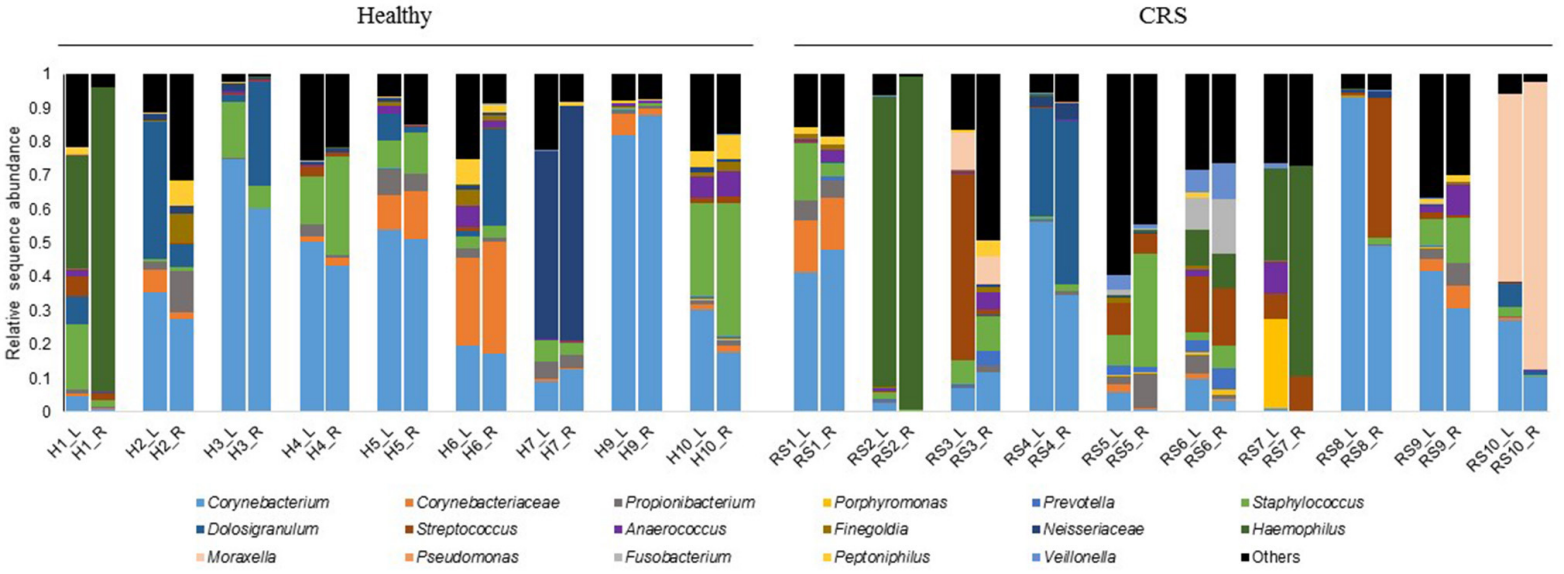
Bacterial community composition of the left (L) and right (R) middle meatus from individuals with CRS and healthy controls. Each color represents a different bacterial genus or family that dominated in the samples. Sequences that do not associate with the dominant genera/families are grouped into “Others.” Relative abundances of 16S rRNA gene sequences are displayed for each sample. L is for left and R is for right middle meatus, H is for healthy controls and RS is for CRS. Patients are labeled 1–10 for the two cohorts, with the exception of patient eight for the healthy cohort, who was not included in this study.

**Figure 5:**
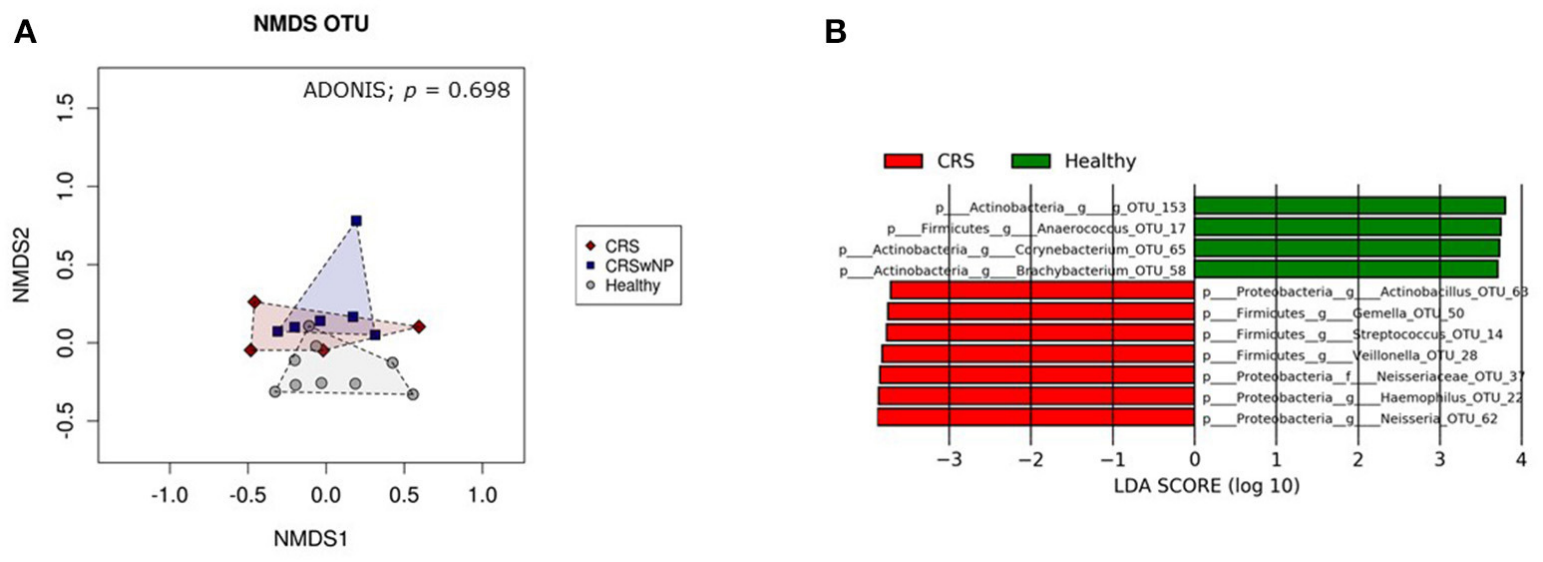
Visualization of bacterial community composition of sinonasal swabs from CRS and healthy individuals. Bacterial 16S rRNA gene data at 97%-OTU level were used to **(A)** construct an nMDS plot, and **(B)** investigate potential microbial biomarkers for CRS and healthy individuals using LDA-LeFSe analysis. The nMDS plot and ADONIS analysis showed no significant difference in bacterial community composition between healthy samples (gray circles) and CRS with polyps (CRSwNP) (blue squares) and without polyps (CRS) (red diamonds). Graphs were made using Calypso software. LDA-LeFSe analysis identified seven potential microbial biomarkers of CRS (colored in red) and four potential biomarkers of heathy samples (colored in green). The taxa identified in this plot differed significantly between the two cohorts of this study. The greater the LDA score, the higher the magnitude of difference (reflecting marked differences in abundance) between cohorts.

Bacterial community composition was highly variable among individuals within each cohort (Figure 4). The most abundant genera across all patients, CRS and Healthy, were *Corynebacterium, Haemophilus, Moraxella*, and *Staphylococcus*. Intra-patient variation was also observed between the right and left nostrils, without any consistent pattern. Thus, left and right samples from each individual were grouped together for further analysis. Visualization of samples in an nMDS plot showed clustering of samples with no significant differences (ADONIS, *p* = 0.70) between healthy control, CRSsNP and CRSwNP cohorts (Figure 5A). LDA-LEfSe analysis identified five genus-level OTUs that strongly associated with CRS disease: *Streptococcus, Haemophilus, Neisseria, Actinobacillus, Gemella*, and *Veillonella* (Figure 5B).

**Figure 6:**
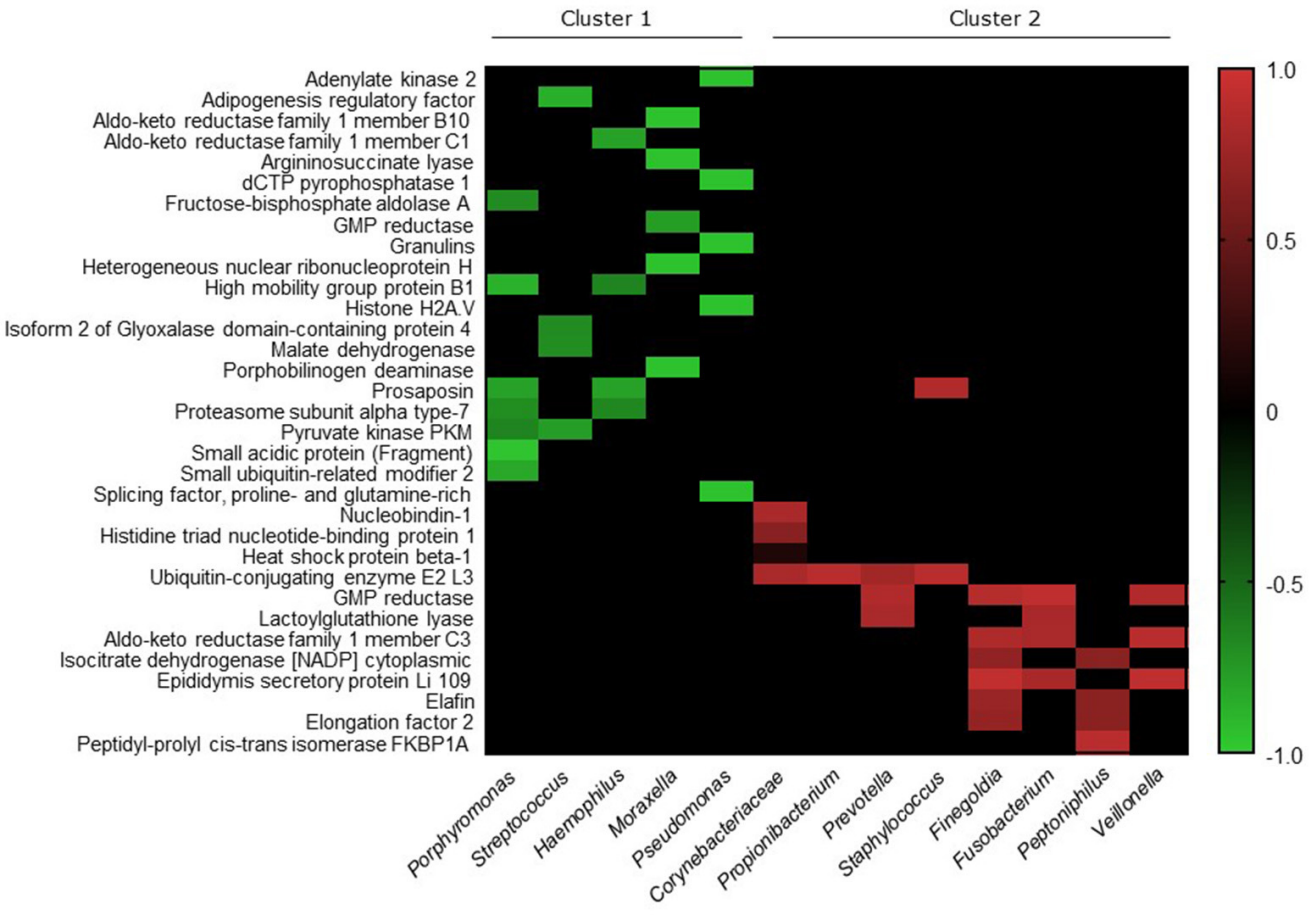
Heatmap depicting the Spearman correlation scores for pairwise comparisons. The top 17 bacteria, with all other bacteria grouped into “Others,” and 104 proteins that were significantly lower in CRS cohort, were analyzed for correlations using Spearman correlation test. Significant correlations were identified between 13 bacterial genera/families and 33 proteins, which are represented in the figure. Ordering of proteins (rows) and bacterial genera/families (columns) were determined using average linkage hierarchical clustering of correlation scores based on Bray Curtis dissimilarity distance matrices. The clusters indicate bacteria and proteins that have more similar correlation scores. Heatmap values are shaded from green (+1) to red (−1) according to correlation scores. Non-significant pairwise associations are colored black.

We investigated correlations between the microbiota data and the seven proteins that were elevated in CRS patients. Significant correlations existed between *Moraxella* and the Ras-related protein (*p* < 0.0001, Spearman *r* = −0.95), and *Corynebacteriaceae* with Angiotensinogen (*p* = 0.024, Spearman *r* = −0.86). Members of the bacterial community with cumulative relative abundance of >80% threshold were chosen for further analysis. Correlations between the lower abundant proteins in CRS was made with these more abundant group of bacteria (Figure 6). Thirty-three out of the 104 proteins with lower abundance in CRS samples correlated significantly (*p* < 0.05) with 13 members of the bacterial community. Two clusters were identified within the bacterial community: one that negatively correlated (r −0.65 to −0.96) and one that positively correlated (r 0.15 to 0.94) with protein abundance.

## Discussion

Multifaceted approaches have been used to understand the dynamic and fundamental interactions between the host and the associated microbiome in multiple systems (Ogilvie and Jones, 2015; Layeghifard et al., 2017; Vorholt et al., 2017). The mucosal layer is highly dynamic and acts as a protective barrier against invading pathogens as well as harboring resident commensal microbiota (Barr et al., 2013). A better understanding of the multifaceted interactions occurring between the host and microbes within this ecosystem will provide insights into mucosal inflammatory diseases such as CRS. In this study, multiple tools were used to elucidate the host-microbe interactions in the sinonasal environment of patients with and without CRS. Immune cells within the underlying tissue were enumerated to describe the inflammatory status of the host. Consistent with previous studies (Van Zele et al., 2006; Kim et al., 2013), our results indicated that the CRS patients had an activated immune response, with increases in T-cells, B-cells and macrophages. The loss of epithelial barrier integrity or reduced expression of tight junction proteins has been argued as a central component in the pathogenesis of CRS (Soyka et al., 2012; Lam et al., 2015). The significant increase in blood proteins in mucus from the CRS cohort found in this study could be another indicator of barrier dysfunction and sinus inflammation. The majority of proteins (82% of the 606 proteins identified) detected in the mucus layer immediately above the mucosa surface did not differ significantly between CRS and healthy individuals. The exact role of these proteins in the sinuses has not been well-described, but we speculate that they could play an important function in physical protection, structure or maintenance. The remaining 18% of all proteins identified (104 lower and 7 higher for CRS individuals) in this study were of great interest and will be discussed further in terms of disease status, the associated bacterial communities and possible clinical implications.

### Proteins associated with sinus disease

Of the seven elevated proteins identified in this study only one protein, S100-A9, has been previously reported as being elevated in CRS patients (Casado et al., 2004; Tewfik et al., 2007; Al Badaai et al., 2009). S100-A9 proteins are important pro-inflammatory mediators that induce chemotaxis of neutrophils and monocytes. In this study they occurred in high abundance and prevalence, supporting their importance in the immune response for CRS patients.

Protein PRRC2C (isoform 3) and 40S ribosomal protein S12 (RPS12) were also more abundant in CRS samples. The associated genes for these two proteins are over expressed in bladder and colorectal cancers, respectively (Huang et al., 2002; Lai and Xu, 2007). The role of RPS12 is to help catalyze protein synthesis, but its exact role in CRS requires further clarification. Protein PRRC2C was elevated >15,000-fold in the CRS group, however, this should be considered with caution as this largely reflects a near-absence of this protein in healthy controls. The function of this protein is to help stem cells differentiate into myeloid or lymphoid lineages of blood cells. We predict that PRRC2C has an active role in driving cell differentiation in the CRS cohort to help combat the chronic nature of this disease.

Other significantly elevated proteins in CRS include Ras-related protein Rab-14, angiotensinogen, low affinity immunoglobulin gamma Fc region receptor Ill-B, and cell division control protein 42 homolog, although all occurred only at low relative abundance (<0.3% of total protein abundance). It is possible that this group of proteins, if expressed before the onset of CRS symptoms, could serve as a biomarker for CRS to help for diagnostic purposes (Das et al., 2008), or for targeted drug therapy.

The significant decrease in abundance of a large number of proteins in the CRS cohort is also of interest. A few of these proteins, TJP2, EZR, granulins, and PYMA, are involved in maintaining epithelial barrier integrity, wound repair, formation of microvilli and mediating immunity to certain types of infections (Bateman and Bennett, 1998; Denker and Nigam, 1998). A loss of epithelial barrier integrity in the sinonasal cavity has long been speculated as a possible cause of CRS. Degradation and lack of mucociliary clearance at the surface is hypothesized to lead to a cascade of reactions that begin with opportunistic microbial infection, movement of microbes into the tissue, a host inflammatory response and ultimately chronic inflammation (Tan et al., 2010). The lower abundance of an important protein required to maintain barrier integrity (TJP2) in the CRS cohort in this study supports the hypothesis that loss of epithelial barrier integrity is important in CRS.

Muc5B and Muc5AC are mucins produced by goblet cells that can be elevated in CRS patients (Ding and Zheng, 2007; Tewfik et al., 2007; Al Badaai et al., 2009; Saieg et al., 2014). However, the results from this study showed no significant differences in Muc5B or Muc5AC protein abundance between the *two* study groups. This could be due to differences in sample sites, sample type, or analytical methods used. It is also possible that with a larger cohort a pattern will become more obvious.

### Phenotypic sub-groups of CRS (CRSwNP and CRSsNP)

Only four proteins differed significantly in abundance between CRS patients with and without nasal polyps. This result should again be interpreted with some caution due to the low sample size within each sub-group. A large number of proteins were eliminated from this analysis as they were not present in three or more samples within each sub-group. Thus, with greater numbers we anticipate the identification of more proteins that can significantly differentiate phenotypic sub-groups of CRS.

Two proteins of particular interest were peptidyl-prolyl cis-trans isomerase A (PPIA) and alcohol dehydrogenase class 4 mu/sigma chain (ADH7), which were significantly higher in CRSsNP and CRSwNP groups, respectively. The PPIA enzyme has been implicated in a wide range of inflammatory and apoptosis pathways, while ADH7 is a hormone that participates in cellular differentiation and could play a crucial role in formation of nasal polyps. The clinical relevance of such findings is discussed below.

### Host-microbe associations

The substantial inter-patient variability in bacterial community structure observed here and elsewhere (Yan et al., 2013; Biswas et al., 2015; Hoggard et al., 2017b) masks obvious patterns between the CRS and healthy control cohorts. As shown in a recent meta-analysis of CRS microbiome datasets, several bacterial phylotypes (analogous to OTUs) were closely associated with disease status (Wagner Mackenzie et al., 2017). A similar approach was applied to the current dataset, identifying certain members of the bacterial community as being strongly associated with disease and health.

This study moves beyond microbial community descriptions, and includes protein data to more completely describe the differences between healthy and CRS individuals at the molecular level. Although a strong negative correlation was observed between *Moraxella* bacteria and Ras-related proteins in this study, due to the low abundance (0.004% of total CRS proteins) of this protein in the CRS cohort the relevance of this finding needs to be validated with more samples. Ras-related proteins are involved in membrane trafficking between Golgi complexes and endosomes during early development. They are also key to basement membrane development. Further investigation will be needed to establish the importance of this protein in CRS disease. Similarly, the nature of the relationship between *Corynebacteriaceae* and angiotensinogen needs to be determined. This protein is a member of a hormone system that regulates blood pressure and fluid balance. In addition, members of the *Corynebacteriaceae* family have been implicated as potential pathogens in CRS (Abreu et al., 2012; Wagner Mackenzie et al., 2017). Thus, the higher abundance of this protein and correlation with a potential pathogen of CRS patients is of interest in the development of targeted drug treatment.

Members of the bacterial genera *Streptococcus* and *Haemophilus* were identified through LDA-LeFSe analysis as having strong associations with CRS. In addition, significant correlative evidence between the lower abundance of certain proteins in CRS cohort with high relative abundance of these two bacteria suggest that they could have an impact on CRS disease progression. While this study shows many novel associations between the host and microbes, it certainly does not completely resolve the etiology of CRS. Research in this area is ongoing and we hope this study will contribute further insights into the complex associations between the host and microbes in the sinuses of diseased and healthy individuals.

### Clinical impact

Proteomics studies have been used successfully to distinguish between patients with CRS and healthy controls (Das et al., 2008; Upton et al., 2011). The application of molecular technologies, including proteomics and bacterial community sequencing, could provide clinicians with molecular bio-profiles for each patient. Such in-depth information has the potential to revolutionize the way in which care is given, in that individualized treatment plans could optimize patient recovery. The results from this study and previous research support the use of proteins as targets for drug therapy or for assessing treatment outcomes in CRS patients.

The results from this study are consistent with those of other studies in CRS, which suggest that this disease is likely to be a result of multiple pathogenic processes (Benninger et al., 2003; Kennedy, 2004; Van Crombruggen et al., 2011) which cause changes to the host tissue and cellular proteome. The focus of this study was on the cellular and microbial interactions of the surface mucosa in the middle meatus, therefore it would be of great interest to investigate whether the patterns identified here are also observed in tissue biopsies.

The routine prescription of antibiotics for CRS treatment at primary care is not recommended, due to the lack of high-quality randomized controlled trials supporting their efficacy (Akkerman et al., 2005), and there is recent evidence that antibiotics may even exacerbate the condition (Maxfield et al., 2017). However, repeated courses are often prescribed. Accordingly, current standard treatment options for CRS need to be re-evaluated and more personalized treatment approaches, perhaps based on technologies such as proteomics, warrant further investigation. A better understanding of the pathophysiology of CRS will be gained through multifaceted studies that provide a more holistic view of the complex host-microbial interactions, and may identify drivers or biomarkers of this disease.

## Summary

Proteomics is an ideal molecular tool for investigating complicated heterogeneous conditions such as CRS. This study showed that 18% of detected proteins (either higher or lower) can help differentiate CRS patients from healthy controls. These proteins, along with associated members of the bacterial community, could be future candidates for biomarkers of CRS. The CRS cohort contained more blood and epithelial or goblet cell proteins and significantly more B-cells and macrophages compared with healthy controls, which is indicative of inflammation. Correlations observed between the host proteins and microbiome, provides a novel insight into host-microbe interactions occurring in the sinonasal cavity of CRS patients. We anticipate that in the future such multifaceted approaches will help establish more effective, personalized treatment options for patients that suffer from CRS.

## Authors contributions

Planned and conceived the study: KB, MT, and RD. Performed the experiment, analyzed the data: KB, BW, SW-T, MM, MJ, and MZ. Wrote the manuscript: KB, Edited the manuscript: KB, BW, SW-T, MM, MJ, MT, and RD.

### Conflict of interest statement

The authors declare that the research was conducted in the absence of any commercial or financial relationships that could be construed as a potential conflict of interest.
