# Supplementary material for: Differentially Regulated Host Proteins Associated with Chronic Rhinosinusitis Are Correlated with the Sinonasal Microbiome
Source: Front Cell Infect Microbiol. 2017 Dec 6;7:504. doi: 10.3389/fcimb.2017.00504 (PMC5723659; doi:10.3389/fcimb.2017.00504)
Supplement: Supplementary file 1 [file DataSheet1.DOCX]

Supplementary Material

**Differentially regulated host proteins associated with chronic rhinosinusitis are correlated with the sinonasal microbiome**

**Kristi Biswas^1^, Brett Wagner Mackenzie^1,2^, Sharon Waldvogel-Thurlow^1^, Martin Middleditch ^2,3^, Mia Jullig^3^, Melissa Zoing^1^, Michael W. Taylor^2,4*^, Richard G. Douglas^1^**

***Correspondence:** Michael W. Taylor: [mw.taylor@auckland.ac.nz](mailto:mw.taylor@auckland.ac.nz)

**Materials and Methods**

*Sample preparation*

The total protein content of each sample was assayed using the EZQ Protein Quantitation Kit (Life Technologies). Aliquots containing 30 µg of protein were reduced by addition of dithiothreitol (DTT) to 10 mM final concentration and incubated at 56°C for 10 min. Samples were cooled and alkylated with 50 mM iodoacetamide (GE Healthcare) in the dark at room temperature for 30 min, then digested with 1 µg of sequencing grade trypsin (Promega) in a chilled microwave (CEM) at 40°C for 2 h at 15W power, followed by overnight incubation at 37°C. Digests were acidified with formic acid and desalted on Oasis HLB SPE cartridges (Waters) and dried down in a vacuum centrifuge. Samples were reconstituted with 30 µL of Dissolution Buffer (Sciex) and labelled with 8-plex iTRAQ labels (Sciex) as per the manufacturer’s instructions. Samples were randomised into three separate pools, each of which also included the same reference sample (a combination of all samples). The combined pools were concentrated in a vacuum centrifuge to reduce solvent content, and then desalted and concentrated to 40 µL as above. Each pool was then fractionated by strong cation-exchange chromatography (SCX) to reduce complexity and improve proteomic coverage during liquid chromatography tandem-mass spectrometry (LC-MS/MS).

*LC-MS/MS Methods*

Samples were processed on three separate mass spectrometry runs. A 2 µL injection was made of each SCX fraction onto a 0.075 x 200 mm picofrit column (New Objective) packed in-house with Reprosil C18 media (Dr Maisch). The following gradient was formed at 250 nL/min using a NanoLC 400 UPLC system (Eksigent): 0-2 min 5%B, 105 min 40%B, 110 min 95%B, 115 min 95%B, 116 min 5%B, 120 min 5%B, where A was 0.1% formic acid in water and B was 0.1% formic acid in acetonitrile.

The picofrit spray was directed into a TripleTOF 6600 Quadrupole-Time-of-Flight mass spectrometer (Sciex) scanning from 350-1600 m/z for 250 ms, followed by 40 ms MS/MS scans on the 40 most abundant multiply-charged peptides (m/z 80-1600) for a total cycle time of ~2 s. The mass spectrometer and HPLC system were under control of the Analyst TF 1.7 software package (Sciex). The resulting data from each pool were searched against a database containing the Uniprot sequences for *Homo sapiens* from February 2015 (89,796 entries) using ProteinPilot version 5.0 (Sciex) with the following parameters: Sample Type, iTRAQ 8-plex (Peptide Labelled); Search Effort, Rapid; Cys Alkylation, Iodoacetamide; Digestion, Trypsin. A “master search” was made by combining the raw data from all pools into a single database search to improve the matching of protein hits across the multiple individual batches. Peptide summaries were exported for further bioinformatic analyses as described below.

*MS/MS data analysis*

The peptide summary files were processed as previously described ([Xu et al., 2015](#_ENREF_46)). Briefly, information on relative abundance was retrieved for each spectrum from individual searches of the three MS experiments. A combined search using the information from all three runs was then conducted, to align protein names and accession numbers for all spectra across experiments. The combined search and the individual searches were linked using acquisition times. The ProteinPilot false discovery report established that any proteins identified with an Unused score <0.7 in the combined search were unreliable (FDR>1%) and these were pruned off, along with spectra associated with insufficient iTRAQ labelling or other disqualifying features. A total of 5729 spectra remained after the trimming step, with these used as a database to graft aligned protein names and accession numbers onto the spectra of the three individual runs, carrying the critical information of the labelling intensity for each separate sample. The link between the overall spectra database and individual sample search was made through the acquisition time. For inclusion in the final analyses, proteins had to be identified in at least three samples (patients) from healthy (max n = 9) or CRS (max n = 10) cohorts. Average ratios within and between the CRS and healthy control groups were calculated for each protein on log-transformed data and a two-tailed t-test was applied to the final ratios.

Further analysis was undertaken to determine the cellular and tissue origin of the identified proteins. For this approach, the total area of all spectra in the 19 samples was calculated to give a total signal (100%). Individual proteins per sample were then assigned a % value of the total. Next, samples were grouped based on disease status (with or without CRS) and an average % for each protein was obtained. The Bgee expression database (<http://bgee.org>) and UniProt database (<http://www.uniprot.org>) were used to locate the origin of the proteins. The four main categories chosen were blood, epithelial/goblet cells, immune cells and other non-specified regions. Proteins were categorised into these groups only if they were highly expressed within one, but not more than one, of these sources.

Healthy

CRS

**Supplementary Figure 1:** Immune cell data for individual patients in CRS and healthy cohort. Standard deviation is shown in the figure.

**Supplementary Table 1:** List of 104 lower abundant proteins in individuals with CRS compared with healthy individuals.

| **# protein** | **Category** | **Accession** | **Name** | | **# H** | **#CRS** | **Healthy Avg** | **CRS Avg** | **LIN CRS/H** | **p (two-tailed t-test)** |
| --- | --- | --- | --- | --- | --- | --- | --- | --- | --- | --- |
|  | **Glycolysis** | |  |  |  |  |  |  |  |  |
| **1** |  | sp\|P19367\|HXK1_HUMAN; sp\|P19367-4\|HXK1_HUMAN; sp\|P19367-3\|HXK1_HUMAN; sp\|P19367-2\|HXK1_HUMAN | **Hexokinase-1 OS=Homo sapiens GN=HK1 PE=1 SV=3; Isoform 4 of Hexokinase-1 OS=Homo sapiens GN=HK1; Isoform 3 of Hexokinase-1 OS=Homo sapiens GN=HK1; Isoform 2 of Hexokinase-1 OS=Homo sapiens GN=HK1** | | 3 | 4 | 1.428 | 0.013 | 0.243 | 0.044 |
| **2** |  | sp\|P04075\|ALDOA_HUMAN; sp\|P04075-2\|ALDOA_HUMAN | **Fructose-bisphosphate aldolase A OS=Homo sapiens GN=ALDOA PE=1 SV=2; Isoform 2 of Fructose-bisphosphate aldolase A OS=Homo sapiens GN=ALDOA** | | 9 | 10 | 0.688 | 0.037 | 0.521 | 0.004 |
| **3** |  | sp\|P09972\|ALDOC_HUMAN | **Fructose-bisphosphate aldolase C OS=Homo sapiens GN=ALDOC PE=1 SV=2** | | 3 | 4 | 2.651 | 1.609 | 0.353 | 0.045 |
| **4** |  | sp\|P14618\|KPYM_HUMAN | **Pyruvate kinase PKM OS=Homo sapiens GN=PKM PE=1 SV=4** | | 9 | 10 | 0.126 | -0.389 | 0.598 | 0.029 |
|  | **Misc carbohydrate metabolism** | | | | | | |  |  |  |
| **5** |  | sp\|P36871\|PGM1_HUMAN | **Phosphoglucomutase-1 OS=Homo sapiens GN=PGM1 PE=1 SV=3** | | 3 | 4 | 0.010 | -0.952 | 0.382 | 0.037 |
|  | **Pentose phosphate shunt** | | | | | | |  |  |  |
| **6** |  | sp\|O95336\|6PGL_HUMAN | **6-phosphogluconolactonase OS=Homo sapiens GN=PGLS PE=1 SV=2** | | 3 | 3 | 0.544 | -0.491 | 0.355 | 0.033 |
|  | **Ketone body metabolism** | |  |  |  |  |  |  |  |  |
| **7** |  | sp\|Q01581\|HMCS1_HUMAN | **Hydroxymethylglutaryl-CoA synthase, cytoplasmic OS=Homo sapiens GN=HMGCS1 PE=1 SV=2** | | 3 | 4 | 0.664 | -0.760 | 0.241 | 0.045 |
|  | **Misc Amino acid metabolism** | | | | | | |  |  |  |
| **8** |  | sp\|P04424\|ARLY_HUMAN; sp\|P04424-3\|ARLY_HUMAN; sp\|P04424-2\|ARLY_HUMAN | **Argininosuccinate lyase OS=Homo sapiens GN=ASL PE=1 SV=4; Isoform 3 of Argininosuccinate lyase OS=Homo sapiens GN=ASL; Isoform 2 of Argininosuccinate lyase OS=Homo sapiens GN=ASL** | | 3 | 4 | 0.921 | -1.005 | 0.146 | 0.019 |
|  | **mt FAO - polyunsaturated etc** | | | | | | |  |  |  |
| **9** |  | sp\|Q16698\|DECR_HUMAN; sp\|Q16698-2\|DECR_HUMAN | **2,4-dienoyl-CoA reductase, mitochondrial OS=Homo sapiens GN=DECR1 PE=1 SV=1; Isoform 2 of 2,4-dienoyl-CoA reductase, mitochondrial OS=Homo sapiens GN=DECR1** | | 3 | 4 | 4.617 | 0.342 | 0.014 | 0.038 |
|  | **Sphingosine metabolism & signalling** | | | | | | |  |  |  |
| **10** |  | tr\|C9JIZ6\|C9JIZ6_HUMAN; tr\|B1AVU8\|B1AVU8_HUMAN; sp\|P07602\|SAP_HUMAN; sp\|P07602-3\|SAP_HUMAN; sp\|P07602-2\|SAP_HUMAN | **Prosaposin OS=Homo sapiens GN=PSAP PE=1 SV=2; Saposin-D OS=Homo sapiens GN=PSAP PE=1 SV=1; Prosaposin OS=Homo sapiens GN=PSAP PE=1 SV=2; Isoform Sap-mu-9 of Prosaposin OS=Homo sapiens GN=PSAP; Isoform Sap-mu-6 of Prosaposin OS=Homo sapiens GN=PSAP** | | 6 | 7 | 0.301 | -0.239 | 0.583 | 0.035 |
|  | **Misc Lipid and steroid metabolism** | | | | | | |  |  |  |
| **11** |  | sp\|Q9BWD1\|THIC_HUMAN; sp\|Q9BWD1-2\|THIC_HUMAN | **Acetyl-CoA acetyltransferase, cytosolic OS=Homo sapiens GN=ACAT2 PE=1 SV=2; Isoform 2 of Acetyl-CoA acetyltransferase, cytosolic OS=Homo sapiens GN=ACAT2** | | 3 | 3 | 2.002 | 0.671 | 0.264 | 0.048 |
| **12** |  | sp\|Q9BX68\|HINT2_HUMAN | **Histidine triad nucleotide-binding protein 2, mitochondrial OS=Homo sapiens GN=HINT2 PE=1 SV=1** | | 3 | 4 | 1.406 | 0.073 | 0.264 | 0.015 |
| **13** |  | tr\|A0A0A0MT30\|A0A0A0MT30_HUMAN; sp\|Q04828\|AK1C1_HUMAN | **Aldo-keto reductase family 1 member C1 OS=Homo sapiens GN=AKR1C1 PE=4 SV=1; Aldo-keto reductase family 1 member C1 OS=Homo sapiens GN=AKR1C1 PE=1 SV=1** | | 6 | 7 | 0.054 | -0.876 | 0.394 | 0.027 |
| **14** |  | tr\|A0A0A0MSS8\|A0A0A0MSS8_HUMAN; sp\|P42330\|AK1C3_HUMAN | **Aldo-keto reductase family 1 member C3 OS=Homo sapiens GN=AKR1C3 PE=4 SV=1; Aldo-keto reductase family 1 member C3 OS=Homo sapiens GN=AKR1C3 PE=1 SV=4** | | 6 | 7 | 0.268 | -0.860 | 0.324 | 0.008 |
|  | **Biotin and misc vitamin related** | | | | | | |  |  |  |
| **15** |  | tr\|E5RG77\|E5RG77_HUMAN; sp\|O94903\|PROSC_HUMAN | **Proline synthase co-transcribed bacterial homolog protein (Fragment) OS=Homo sapiens GN=PROSC PE=1 SV=1; Proline synthase co-transcribed bacterial homolog protein OS=Homo sapiens GN=PROSC PE=1 SV=1** | | 6 | 7 | 0.949 | -0.612 | 0.210 | 0.008 |
|  | **Krebs cycle** | |  | | | | |  |  |  |
| **16** |  | sp\|P40926\|MDHM_HUMAN | **Malate dehydrogenase, mitochondrial OS=Homo sapiens GN=MDH2 PE=1 SV=3** | | 9 | 10 | 0.837 | 0.101 | 0.479 | 0.025 |
|  | **Iron-sulfur cluster protein related** | | | | | | |  |  |  |
| **17** |  | sp\|P38646\|GRP75_HUMAN | **Stress-70 protein, mitochondrial OS=Homo sapiens GN=HSPA9 PE=1 SV=2** | | 3 | 4 | 1.561 | 0.036 | 0.218 | 0.011 |
|  | **ETS** |  |  | |  |  |  |  |  |  |
| **18** |  | sp\|P25705\|ATPA_HUMAN; sp\|P25705-2\|ATPA_HUMAN | **ATP synthase subunit alpha, mitochondrial OS=Homo sapiens GN=ATP5A1 PE=1 SV=1; Isoform 2 of ATP synthase subunit alpha, mitochondrial OS=Homo sapiens GN=ATP5A1** | | 3 | 4 | 2.523 | -0.273 | 0.061 | 0.025 |
|  | **Creatine kinases** | |  |  |  |  |  |  |  |  |
| **19** |  | sp\|P12277\|KCRB_HUMAN | **Creatine kinase B-type OS=Homo sapiens GN=CKB PE=1 SV=1** | | 9 | 10 | 0.856 | -0.311 | 0.311 | 0.003 |
|  | **Misc nucleotides** | |  |  |  |  |  |  |  |  |
| **20** |  | sp\|P30520\|PURA2_HUMAN | **Adenylosuccinate synthetase isozyme 2 OS=Homo sapiens GN=ADSS PE=1 SV=3** | | 3 | 4 | 3.330 | 0.805 | 0.080 | 0.028 |
| **21** |  | tr\|F8W1A4\|F8W1A4_HUMAN; sp\|P54819\|KAD2_HUMAN; sp\|P54819-2\|KAD2_HUMAN; sp\|P54819-5\|KAD2_HUMAN | **Adenylate kinase 2, mitochondrial OS=Homo sapiens GN=AK2 PE=1 SV=1; Adenylate kinase 2, mitochondrial OS=Homo sapiens GN=AK2 PE=1 SV=2; Isoform 2 of Adenylate kinase 2, mitochondrial OS=Homo sapiens GN=AK2; Isoform 5 of Adenylate kinase 2, mitochondrial OS=Homo sapiens GN=AK2** | | 3 | 4 | 2.718 | 1.135 | 0.205 | 0.004 |
| **22** |  | sp\|Q9H773\|DCTP1_HUMAN | **dCTP pyrophosphatase 1 OS=Homo sapiens GN=DCTPP1 PE=1 SV=1** | | 3 | 4 | 1.413 | -0.626 | 0.130 | 0.005 |
| **23** |  | tr\|H0YNJ6\|H0YNJ6_HUMAN; tr\|H0YMB3\|H0YMB3_HUMAN; tr\|H0YLV5\|H0YLV5_HUMAN; tr\|F8WAN9\|F8WAN9_HUMAN; tr\|A0A087WWM4\|A0A087WWM4_HUMAN; sp\|Q9P2T1\|GMPR2_HUMAN; sp\|Q9P2T1-2\|GMPR2_HUMAN | **GMP reductase OS=Homo sapiens GN=GMPR2 PE=3 SV=1; GMP reductase 2 OS=Homo sapiens GN=GMPR2 PE=1 SV=1; GMP reductase 2 OS=Homo sapiens GN=GMPR2 PE=1 SV=1; GMP reductase OS=Homo sapiens GN=GMPR2 PE=1 SV=1; GMP reductase OS=Homo sapiens GN=GMPR2 PE=1 SV=1; GMP reductase 2 OS=Homo sapiens GN=GMPR2 PE=1 SV=1; Isoform 2 of GMP reductase 2 OS=Homo sapiens GN=GMPR2** | | 6 | 7 | 0.884 | -0.280 | 0.312 | 0.027 |
|  | **Porphyrin metabolism** | |  |  |  |  |  |  |  |  |
| **24** |  | tr\|F5H345\|F5H345_HUMAN; sp\|P08397\|HEM3_HUMAN; sp\|P08397-4\|HEM3_HUMAN; sp\|P08397-3\|HEM3_HUMAN; sp\|P08397-2\|HEM3_HUMAN | **Porphobilinogen deaminase OS=Homo sapiens GN=HMBS PE=1 SV=1; Porphobilinogen deaminase OS=Homo sapiens GN=HMBS PE=1 SV=2; Isoform 4 of Porphobilinogen deaminase OS=Homo sapiens GN=HMBS; Isoform 3 of Porphobilinogen deaminase OS=Homo sapiens GN=HMBS; Isoform 2 of Porphobilinogen deaminase OS=Homo sapiens GN=HMBS** | | 3 | 4 | 1.299 | 0.055 | 0.288 | 0.045 |
|  | **AMINO SUGARS AND NUCLEOTIDE SUGARS** | | | | | | |  |  |  |
| **25** |  | sp\|Q9NR45\|SIAS_HUMAN | **Sialic acid synthase OS=Homo sapiens GN=NANS PE=1 SV=2** | | 6 | 7 | 0.424 | -0.528 | 0.386 | 0.027 |
|  | **Misc metabolism** | |  | | | | |  |  |  |
| **26** |  | sp\|P30838\|AL3A1_HUMAN | **Aldehyde dehydrogenase, dimeric NADP-preferring OS=Homo sapiens GN=ALDH3A1 PE=1 SV=3** | | 9 | 10 | -0.106 | -1.033 | 0.396 | 0.019 |
| **27** |  | sp\|P00326\|ADH1G_HUMAN | **Alcohol dehydrogenase 1C OS=Homo sapiens GN=ADH1C PE=1 SV=2** | | 9 | 10 | 0.054 | -0.897 | 0.387 | 0.024 |
| **28** |  | sp\|O60218\|AK1BA_HUMAN | **Aldo-keto reductase family 1 member B10 OS=Homo sapiens GN=AKR1B10 PE=1 SV=2** | | 3 | 4 | 0.241 | -1.237 | 0.228 | 0.004 |
| **29** |  | sp\|O75874\|IDHC_HUMAN | **Isocitrate dehydrogenase [NADP] cytoplasmic OS=Homo sapiens GN=IDH1 PE=1 SV=2** | | 9 | 10 | 1.331 | 0.180 | 0.316 | 0.001 |
| **30** |  | tr\|H0YLA4\|H0YLA4_HUMAN; sp\|Q00796\|DHSO_HUMAN | **Sorbitol dehydrogenase OS=Homo sapiens GN=SORD PE=1 SV=1; Sorbitol dehydrogenase OS=Homo sapiens GN=SORD PE=1 SV=4** | | 9 | 10 | 0.381 | -0.619 | 0.368 | 0.026 |
| **31** |  | tr\|C9JH92\|C9JH92_HUMAN; tr\|A6NP24\|A6NP24_HUMAN; sp\|Q08257\|QOR_HUMAN; sp\|Q08257-3\|QOR_HUMAN | **Quinone oxidoreductase (Fragment) OS=Homo sapiens GN=CRYZ PE=1 SV=1; Quinone oxidoreductase (Fragment) OS=Homo sapiens GN=CRYZ PE=1 SV=1; Quinone oxidoreductase OS=Homo sapiens GN=CRYZ PE=1 SV=1; Isoform 3 of Quinone oxidoreductase OS=Homo sapiens GN=CRYZ** | | 3 | 4 | 1.731 | -0.667 | 0.091 | 0.004 |
|  | **Misc ECM/Adhesion** | |  | | | | |  |  |  |
| **32** |  | sp\|Q9UDY2\|ZO2_HUMAN; sp\|Q9UDY2-7\|ZO2_HUMAN; sp\|Q9UDY2-6\|ZO2_HUMAN; sp\|Q9UDY2-3\|ZO2_HUMAN | **Tight junction protein ZO-2 OS=Homo sapiens GN=TJP2 PE=1 SV=2; Isoform 7 of Tight junction protein ZO-2 OS=Homo sapiens GN=TJP2; Isoform 6 of Tight junction protein ZO-2 OS=Homo sapiens GN=TJP2; Isoform C1 of Tight junction protein ZO-2 OS=Homo sapiens GN=TJP2** | | 3 | 4 | 1.254 | -0.459 | 0.180 | 0.005 |
|  | **Bacteriolysis, immune function, inflammatory response** | | | | | | |  |  |  |
| **33** |  | tr\|H7C2N1\|H7C2N1_HUMAN; tr\|B8ZZW7\|B8ZZW7_HUMAN; tr\|B8ZZQ6\|B8ZZQ6_HUMAN; tr\|B8ZZA1\|B8ZZA1_HUMAN; sp\|P06454\|PTMA_HUMAN; sp\|P06454-2\|PTMA_HUMAN | **Prothymosin alpha (Fragment) OS=Homo sapiens GN=PTMA PE=1 SV=1; Prothymosin alpha OS=Homo sapiens GN=PTMA PE=1 SV=1; Prothymosin alpha OS=Homo sapiens GN=PTMA PE=1 SV=1; Prothymosin alpha OS=Homo sapiens GN=PTMA PE=1 SV=1; Prothymosin alpha OS=Homo sapiens GN=PTMA PE=1 SV=2; Isoform 2 of Prothymosin alpha OS=Homo sapiens GN=PTMA** | | 9 | 10 | 1.344 | 0.565 | 0.459 | 0.036 |
| **34** |  | sp\|P28799\|GRN_HUMAN; sp\|P28799-2\|GRN_HUMAN | **Granulins OS=Homo sapiens GN=GRN PE=1 SV=2; Isoform 2 of Granulins OS=Homo sapiens GN=GRN** | | 3 | 4 | 2.338 | 0.697 | 0.194 | 0.025 |
| **35** |  | sp\|P09341\|GROA_HUMAN | **Growth-regulated alpha protein OS=Homo sapiens GN=CXCL1 PE=1 SV=1** | | 3 | 4 | 2.524 | 0.700 | 0.161 | 0.008 |
|  | **Apolipoproteins** | |  | | | | |  |  |  |
| **36** |  | sp\|P10909\|CLUS_HUMAN; sp\|P10909-5\|CLUS_HUMAN; sp\|P10909-4\|CLUS_HUMAN; sp\|P10909-2\|CLUS_HUMAN | **Clusterin OS=Homo sapiens GN=CLU PE=1 SV=1; Isoform 5 of Clusterin OS=Homo sapiens GN=CLU; Isoform 4 of Clusterin OS=Homo sapiens GN=CLU; Isoform 2 of Clusterin OS=Homo sapiens GN=CLU** | | 6 | 7 | 1.405 | 0.539 | 0.421 | 0.017 |
|  | **ACTIN/cytoskeleton related** | | | | | | |  |  |  |
| **37** |  | sp\|O14745\|NHRF1_HUMAN | **Na(+)/H(+) exchange regulatory cofactor NHE-RF1 OS=Homo sapiens GN=SLC9A3R1 PE=1 SV=4** | | 6 | 7 | 1.831 | 0.818 | 0.363 | 0.011 |
| **38** |  | tr\|E7EQR4\|E7EQR4_HUMAN; sp\|P15311\|EZRI_HUMAN | **Ezrin OS=Homo sapiens GN=EZR PE=1 SV=3; Ezrin OS=Homo sapiens GN=EZR PE=1 SV=4** | | 9 | 10 | 0.913 | 0.013 | 0.407 | 0.002 |
| **39** |  | sp\|Q9NYL9\|TMOD3_HUMAN | **Tropomodulin-3 OS=Homo sapiens GN=TMOD3 PE=1 SV=1** | | 3 | 4 | 0.928 | -0.138 | 0.344 | 0.006 |
|  | **Misc Golgi** | |  | | | | |  |  |  |
| **40** |  | sp\|Q02818\|NUCB1_HUMAN | **Nucleobindin-1 OS=Homo sapiens GN=NUCB1 PE=1 SV=4** | | 6 | 7 | 1.868 | 0.701 | 0.311 | 0.039 |
|  | **Misc lysosomes** | |  | | | | |  |  |  |
| **41** |  | tr\|V9HW75\|V9HW75_HUMAN | **Epididymis secretory protein Li 109 OS=Homo sapiens GN=HEL-S-109 PE=2 SV=1** | | 6 | 7 | 0.370 | -0.369 | 0.477 | 0.049 |
|  | **Misc mitochondrial** | |  | | | | |  |  |  |
| **42** |  | sp\|P49773\|HINT1_HUMAN | **Histidine triad nucleotide-binding protein 1 OS=Homo sapiens GN=HINT1 PE=1 SV=2** | | 9 | 10 | 1.306 | 0.397 | 0.403 | 0.017 |
| **43** |  | sp\|Q9HC38-2\|GLOD4_HUMAN | **Isoform 2 of Glyoxalase domain-containing protein 4 OS=Homo sapiens GN=GLOD4** | | 9 | 10 | 0.765 | -0.075 | 0.432 | 0.006 |
|  | **Superoxide/peroxide/nitric oxide related** | | | | | | |  |  |  |
| **44** |  | sp\|Q04760\|LGUL_HUMAN | **Lactoylglutathione lyase OS=Homo sapiens GN=GLO1 PE=1 SV=4** | | 6 | 7 | 1.988 | -0.133 | 0.120 | 0.031 |
|  | **thioredoxin domain containing and Thioredoxin-related transmembrane protein** | | | | | | |  |  |  |
| **45** |  | sp\|O95881\|TXD12_HUMAN | **Thioredoxin domain-containing protein 12 OS=Homo sapiens GN=TXNDC12 PE=1 SV=1** | | 3 | 4 | 6.747 | 3.673 | 0.046 | 0.041 |
|  | **Detox - Carboxylesterases** | | | | | | |  |  |  |
| **46** |  | sp\|P23141\|EST1_HUMAN; sp\|P23141-3\|EST1_HUMAN; sp\|P23141-2\|EST1_HUMAN | **Liver carboxylesterase 1 OS=Homo sapiens GN=CES1 PE=1 SV=2; Isoform 3 of Liver carboxylesterase 1 OS=Homo sapiens GN=CES1; Isoform 2 of Liver carboxylesterase 1 OS=Homo sapiens GN=CES1** | | 9 | 9 | 0.706 | -0.814 | 0.219 | 0.004 |
|  | **Glutathione S-transferase** | | | | | | |  |  |  |
| **47** |  | sp\|P78417\|GSTO1_HUMAN | **Glutathione S-transferase omega-1 OS=Homo sapiens GN=GSTO1 PE=1 SV=2** | | 6 | 7 | 0.238 | -0.565 | 0.448 | 0.010 |
|  | **chaperonin containing Tcp1 - folds tubulin and actin** | | | | | | |  |  |  |
| **48** |  | sp\|Q99832\|TCPH_HUMAN | **T-complex protein 1 subunit eta OS=Homo sapiens GN=CCT7 PE=1 SV=2** | | 3 | 4 | 1.011 | -0.647 | 0.190 | 0.049 |
|  | **Misc Heat shock/chaperone** | | | | | | |  |  |  |
| **49** |  | sp\|P10809\|CH60_HUMAN | **60 kDa heat shock protein, mitochondrial OS=Homo sapiens GN=HSPD1 PE=1 SV=2** | | 6 | 7 | 1.275 | 0.478 | 0.451 | 0.032 |
| **50** |  | sp\|P04792\|HSPB1_HUMAN | **Heat shock protein beta-1 OS=Homo sapiens GN=HSPB1 PE=1 SV=2** | | 9 | 10 | 1.335 | 0.480 | 0.425 | 0.021 |
| **51** |  | sp\|P08238\|HS90B_HUMAN | **Heat shock protein HSP 90-beta OS=Homo sapiens GN=HSP90AB1 PE=1 SV=4** | | 6 | 7 | 0.324 | -0.546 | 0.419 | 0.025 |
| **52** |  | sp\|P50502\|F10A1_HUMAN | **Hsc70-interacting protein OS=Homo sapiens GN=ST13 PE=1 SV=2** | | 6 | 7 | 0.860 | -0.123 | 0.374 | 0.043 |
| **53** |  | sp\|P14625\|ENPL_HUMAN | **Endoplasmin OS=Homo sapiens GN=HSP90B1 PE=1 SV=1** | | 6 | 7 | 1.597 | 0.436 | 0.313 | 0.039 |
| **54** |  | sp\|Q15084\|PDIA6_HUMAN; sp\|Q15084-5\|PDIA6_HUMAN; sp\|Q15084-4\|PDIA6_HUMAN; sp\|Q15084-3\|PDIA6_HUMAN; sp\|Q15084-2\|PDIA6_HUMAN | **Protein disulfide-isomerase A6 OS=Homo sapiens GN=PDIA6 PE=1 SV=1; Isoform 5 of Protein disulfide-isomerase A6 OS=Homo sapiens GN=PDIA6; Isoform 4 of Protein disulfide-isomerase A6 OS=Homo sapiens GN=PDIA6; Isoform 3 of Protein disulfide-isomerase A6 OS=Homo sapiens GN=PDIA6; Isoform 2 of Protein disulfide-isomerase A6 OS=Homo sapiens GN=PDIA6** | | 3 | 4 | 1.013 | -0.224 | 0.290 | 0.001 |
|  | **Kinases** | |  | | | | |  |  |  |
| **55** |  | sp\|Q9H1E3\|NUCKS_HUMAN; sp\|Q9H1E3-2\|NUCKS_HUMAN | **Nuclear ubiquitous casein and cyclin-dependent kinase substrate 1 OS=Homo sapiens GN=NUCKS1 PE=1 SV=1; Isoform 2 of Nuclear ubiquitous casein and cyclin-dependent kinase substrate 1 OS=Homo sapiens GN=NUCKS1** | | 3 | 4 | 2.776 | 0.983 | 0.166 | 0.007 |
|  | **Phosphatases** | |  | | | | |  |  |  |
| **56** |  | sp\|P35813\|PPM1A_HUMAN; sp\|P35813-3\|PPM1A_HUMAN | **Protein phosphatase 1A OS=Homo sapiens GN=PPM1A PE=1 SV=1; Isoform 3 of Protein phosphatase 1A OS=Homo sapiens GN=PPM1A** | | 3 | 4 | 1.427 | -1.626 | 0.047 | 0.045 |
|  | **Chloride intracellular channel** | | | | | | |  |  |  |
| **57** |  | sp\|O00299\|CLIC1_HUMAN | **Chloride intracellular channel protein 1 OS=Homo sapiens GN=CLIC1 PE=1 SV=4** | | 6 | 7 | -0.093 | -0.900 | 0.446 | 0.032 |
|  | **Misc metal binding/homeostasis** | | | | | | |  |  |  |
| **58** |  | sp\|Q8N339\|MT1M_HUMAN; sp\|P80297\|MT1X_HUMAN; sp\|P13640\|MT1G_HUMAN; sp\|P13640-2\|MT1G_HUMAN; sp\|P04732\|MT1E_HUMAN; sp\|P02795\|MT2_HUMAN | **Metallothionein-1M OS=Homo sapiens GN=MT1M PE=2 SV=2; Metallothionein-1X OS=Homo sapiens GN=MT1X PE=1 SV=1; Metallothionein-1G OS=Homo sapiens GN=MT1G PE=1 SV=2; Isoform 2 of Metallothionein-1G OS=Homo sapiens GN=MT1G; Metallothionein-1E OS=Homo sapiens GN=MT1E PE=1 SV=1; Metallothionein-2 OS=Homo sapiens GN=MT2A PE=1 SV=1** | | 3 | 4 | 0.973 | 0.081 | 0.410 | 0.000 |
|  | **Glucosidases** | |  | | | | |  |  |  |
| **59** |  | sp\|Q14697\|GANAB_HUMAN; sp\|Q14697-2\|GANAB_HUMAN | **Neutral alpha-glucosidase AB OS=Homo sapiens GN=GANAB PE=1 SV=3; Isoform 2 of Neutral alpha-glucosidase AB OS=Homo sapiens GN=GANAB** | | 3 | 4 | 0.575 | -0.387 | 0.382 | 0.018 |
|  | **Ubiquitination** | |  | | | | |  |  |  |
| **60** |  | sp\|P61956\|SUMO2_HUMAN; sp\|P61956-2\|SUMO2_HUMAN | **Small ubiquitin-related modifier 2 OS=Homo sapiens GN=SUMO2 PE=1 SV=3; Isoform 2 of Small ubiquitin-related modifier 2 OS=Homo sapiens GN=SUMO2** | | 6 | 7 | 0.905 | -0.043 | 0.387 | 0.014 |
| **61** |  | sp\|P68036\|UB2L3_HUMAN; sp\|P68036-3\|UB2L3_HUMAN; sp\|P68036-2\|UB2L3_HUMAN | **Ubiquitin-conjugating enzyme E2 L3 OS=Homo sapiens GN=UBE2L3 PE=1 SV=1; Isoform 3 of Ubiquitin-conjugating enzyme E2 L3 OS=Homo sapiens GN=UBE2L3; Isoform 2 of Ubiquitin-conjugating enzyme E2 L3 OS=Homo sapiens GN=UBE2L3** | | 6 | 7 | 1.770 | 0.687 | 0.339 | 0.037 |
|  | **Proteasome** | |  | | | | |  |  |  |
| **62** |  | tr\|G3V5Z7\|G3V5Z7_HUMAN; sp\|P60900\|PSA6_HUMAN; sp\|P60900-2\|PSA6_HUMAN | **Proteasome subunit alpha type OS=Homo sapiens GN=PSMA6 PE=1 SV=1; Proteasome subunit alpha type-6 OS=Homo sapiens GN=PSMA6 PE=1 SV=1; Isoform 2 of Proteasome subunit alpha type-6 OS=Homo sapiens GN=PSMA6** | | 6 | 7 | 1.088 | 0.361 | 0.483 | 0.020 |
| **63** |  | sp\|O14818\|PSA7_HUMAN | **Proteasome subunit alpha type-7 OS=Homo sapiens GN=PSMA7 PE=1 SV=1** | | 9 | 10 | 1.089 | -0.783 | 0.154 | 0.046 |
| **64** |  | sp\|P40306\|PSB10_HUMAN | **Proteasome subunit beta type-10 OS=Homo sapiens GN=PSMB10 PE=1 SV=1** | | 3 | 4 | 0.584 | -0.495 | 0.340 | 0.025 |
|  | **Protease inhibitors** | |  | | | | |  |  |  |
| **65** |  | tr\|E9PCH5\|E9PCH5_HUMAN; tr\|E7EVY3\|E7EVY3_HUMAN; tr\|E7ES10\|E7ES10_HUMAN; tr\|B7Z574\|B7Z574_HUMAN; sp\|P20810\|ICAL_HUMAN; sp\|P20810-8\|ICAL_HUMAN; sp\|P20810-6\|ICAL_HUMAN; sp\|P20810-5\|ICAL_HUMAN; sp\|P20810-2\|ICAL_HUMAN; sp\|P20810-10\|ICAL_HUMAN | **Calpastatin OS=Homo sapiens GN=CAST PE=1 SV=1; Calpastatin OS=Homo sapiens GN=CAST PE=1 SV=1; Calpastatin (Fragment) OS=Homo sapiens GN=CAST PE=1 SV=1; Calpastatin OS=Homo sapiens GN=CAST PE=2 SV=1; Calpastatin OS=Homo sapiens GN=CAST PE=1 SV=4; Isoform 8 of Calpastatin OS=Homo sapiens GN=CAST; Isoform 6 of Calpastatin OS=Homo sapiens GN=CAST; Isoform 5 of Calpastatin OS=Homo sapiens GN=CAST; Isoform 2 of Calpastatin OS=Homo sapiens GN=CAST; Isoform 10 of Calpastatin OS=Homo sapiens GN=CAST** | | 6 | 7 | 0.692 | -0.013 | 0.494 | 0.038 |
| **66** |  | sp\|P19957\|ELAF_HUMAN | **Elafin OS=Homo sapiens GN=PI3 PE=1 SV=3** | | 9 | 10 | 0.572 | -0.480 | 0.349 | 0.020 |
|  | **nuclear export of spliced and unspliced mRNA** | | | | | | |  |  |  |
| **67** |  | tr\|E9PB61\|E9PB61_HUMAN; sp\|Q86V81\|THOC4_HUMAN | **THO complex subunit 4 OS=Homo sapiens GN=ALYREF PE=1 SV=1; THO complex subunit 4 OS=Homo sapiens GN=ALYREF PE=1 SV=3** | | 3 | 4 | 2.917 | 0.457 | 0.085 | 0.002 |
|  | **Endocytosis** | |  | | | | |  |  |  |
| **68** |  | sp\|P09496\|CLCA_HUMAN; sp\|P09496-4\|CLCA_HUMAN; sp\|P09496-3\|CLCA_HUMAN; sp\|P09496-2\|CLCA_HUMAN | **Clathrin light chain A OS=Homo sapiens GN=CLTA PE=1 SV=1; Isoform 4 of Clathrin light chain A OS=Homo sapiens GN=CLTA; Isoform 3 of Clathrin light chain A OS=Homo sapiens GN=CLTA; Isoform Non-brain of Clathrin light chain A OS=Homo sapiens GN=CLTA** | | 3 | 4 | 0.469 | -0.226 | 0.499 | 0.037 |
|  | **G protein coupled receptors related** | | | | | | |  |  |  |
| **69** |  | sp\|P49407\|ARRB1_HUMAN; sp\|P49407-2\|ARRB1_HUMAN | **Beta-arrestin-1 OS=Homo sapiens GN=ARRB1 PE=1 SV=2; Isoform 1B of Beta-arrestin-1 OS=Homo sapiens GN=ARRB1** | | 3 | 4 | 1.148 | -0.391 | 0.215 | 0.005 |
|  | **Rho - cytoskeletal dynamics/morphology** | | | | | | |  |  |  |
| **70** |  | sp\|P63000\|RAC1_HUMAN; sp\|P63000-2\|RAC1_HUMAN | **Ras-related C3 botulinum toxin substrate 1 OS=Homo sapiens GN=RAC1 PE=1 SV=1; Isoform B of Ras-related C3 botulinum toxin substrate 1 OS=Homo sapiens GN=RAC1** | | 6 | 7 | 1.092 | -0.228 | 0.267 | 0.011 |
|  | **Rab - membrane trafficking** | | | | | | |  |  |  |
| **71** |  | sp\|Q9H0U4\|RAB1B_HUMAN | **Ras-related protein Rab-1B OS=Homo sapiens GN=RAB1B PE=1 SV=1** | | 3 | 4 | 0.065 | -1.106 | 0.310 | 0.030 |
|  | **Ran - nuclear transport** | |  | | | | |  |  |  |
| **72** |  | tr\|F6WQW2\|F6WQW2_HUMAN; tr\|C9JXG8\|C9JXG8_HUMAN; sp\|P43487\|RANG_HUMAN; sp\|P43487-2\|RANG_HUMAN | **Ran-specific GTPase-activating protein OS=Homo sapiens GN=RANBP1 PE=1 SV=1; Ran-specific GTPase-activating protein (Fragment) OS=Homo sapiens GN=RANBP1 PE=1 SV=2; Ran-specific GTPase-activating protein OS=Homo sapiens GN=RANBP1 PE=1 SV=1; Isoform 2 of Ran-specific GTPase-activating protein OS=Homo sapiens GN=RANBP1** | | 3 | 4 | 2.012 | -0.213 | 0.108 | 0.017 |
|  | **Misc Cell signalling** | |  | | | | |  |  |  |
| **73** |  | tr\|D6RHD7\|D6RHD7_HUMAN; tr\|D6RF97\|D6RF97_HUMAN; sp\|Q8WWF8\|CAPSL_HUMAN | **Calcyphosin-like protein OS=Homo sapiens GN=CAPSL PE=4 SV=1; Calcyphosin-like protein (Fragment) OS=Homo sapiens GN=CAPSL PE=4 SV=1; Calcyphosin-like protein OS=Homo sapiens GN=CAPSL PE=2 SV=4** | | 3 | 3 | 2.046 | 0.635 | 0.244 | 0.017 |
| **74** |  | sp\|Q71UI9\|H2AV_HUMAN; sp\|P0C0S5\|H2AZ_HUMAN | **Histone H2A.V OS=Homo sapiens GN=H2AFV PE=1 SV=3; Histone H2A.Z OS=Homo sapiens GN=H2AFZ PE=1 SV=2** | | 3 | 4 | 2.530 | 0.054 | 0.084 | 0.001 |
|  | **nucleosome and chromatin** | | | | | | |  |  |  |
| **75** |  | sp\|P09429\|HMGB1_HUMAN | **High mobility group protein B1 OS=Homo sapiens GN=HMGB1 PE=1 SV=3** | | 9 | 10 | 0.706 | 0.213 | 0.611 | 0.015 |
|  | **Misc transcription etc** | |  | | | | |  |  |  |
| **76** |  | sp\|O75475\|PSIP1_HUMAN | **PC4 and SFRS1-interacting protein OS=Homo sapiens GN=PSIP1 PE=1 SV=1** | | 3 | 4 | 2.692 | 1.209 | 0.227 | 0.004 |
|  | **Ribosomal** | |  | | | | |  |  |  |
| **77** |  | sp\|P62280\|RS11_HUMAN | **40S ribosomal protein S11 OS=Homo sapiens GN=RPS11 PE=1 SV=3** | | 3 | 4 | 1.896 | 0.184 | 0.180 | 0.006 |
| **78** |  | sp\|P46778\|RL21_HUMAN | **60S ribosomal protein L21 OS=Homo sapiens GN=RPL21 PE=1 SV=2** | | 3 | 4 | 1.874 | 0.481 | 0.248 | 0.026 |
| **79** |  | sp\|Q02878\|RL6_HUMAN | **60S ribosomal protein L6 OS=Homo sapiens GN=RPL6 PE=1 SV=3** | | 6 | 7 | 1.806 | 0.052 | 0.173 | 0.011 |
|  | **eukaryotic translation initiation factors and regulators** | | | | | | |  |  |  |
| **80** |  | sp\|P47813\|IF1AX_HUMAN; sp\|O14602\|IF1AY_HUMAN | **Eukaryotic translation initiation factor 1A, X-chromosomal OS=Homo sapiens GN=EIF1AX PE=1 SV=2; Eukaryotic translation initiation factor 1A, Y-chromosomal OS=Homo sapiens GN=EIF1AY PE=1 SV=4** | | 3 | 4 | 1.610 | 0.383 | 0.293 | 0.033 |
| **81** |  | sp\|Q15056\|IF4H_HUMAN; sp\|Q15056-2\|IF4H_HUMAN | **Eukaryotic translation initiation factor 4H OS=Homo sapiens GN=EIF4H PE=1 SV=5; Isoform Short of Eukaryotic translation initiation factor 4H OS=Homo sapiens GN=EIF4H** | | 3 | 4 | 1.059 | -0.317 | 0.253 | 0.014 |
| **82** |  | sp\|P63241-2\|IF5A1_HUMAN | **Isoform 2 of Eukaryotic translation initiation factor 5A-1 OS=Homo sapiens GN=EIF5A** | | 9 | 10 | -0.063 | -0.797 | 0.480 | 0.013 |
|  | **nuclear ribonucleoproteins** | | | | | | |  |  |  |
| **83** |  | tr\|G8JLB6\|G8JLB6_HUMAN; tr\|E9PCY7\|E9PCY7_HUMAN; sp\|P55795\|HNRH2_HUMAN; sp\|P31943\|HNRH1_HUMAN | **Heterogeneous nuclear ribonucleoprotein H OS=Homo sapiens GN=HNRNPH1 PE=1 SV=1; Heterogeneous nuclear ribonucleoprotein H OS=Homo sapiens GN=HNRNPH1 PE=1 SV=1; Heterogeneous nuclear ribonucleoprotein H2 OS=Homo sapiens GN=HNRNPH2 PE=1 SV=1; Heterogeneous nuclear ribonucleoprotein H OS=Homo sapiens GN=HNRNPH1 PE=1 SV=4** | | 3 | 4 | 0.589 | -0.467 | 0.348 | 0.041 |
|  | **Splicing factors / helicases** | | | | | | |  |  |  |
| **84** |  | sp\|P23246\|SFPQ_HUMAN; sp\|P23246-2\|SFPQ_HUMAN | **Splicing factor, proline- and glutamine-rich OS=Homo sapiens GN=SFPQ PE=1 SV=2; Isoform Short of Splicing factor, proline- and glutamine-rich OS=Homo sapiens GN=SFPQ** | | 3 | 4 | 1.407 | 0.196 | 0.298 | 0.022 |
|  | **Misc Protein biosynthesis** | | | | | | |  |  |  |
| **85** |  | sp\|P26641-2\|EF1G_HUMAN | **Isoform 2 of Elongation factor 1-gamma OS=Homo sapiens GN=EEF1G** | | 9 | 10 | 0.609 | -0.182 | 0.453 | 0.023 |
| **86** |  | sp\|P13639\|EF2_HUMAN | **Elongation factor 2 OS=Homo sapiens GN=EEF2 PE=1 SV=4** | | 9 | 10 | 0.653 | -0.532 | 0.305 | 0.004 |
|  | **TPD52 family** | |  | | | | |  |  |  |
| **87** |  | tr\|H0YC42\|H0YC42_HUMAN; tr\|F5H0B0\|F5H0B0_HUMAN; sp\|P55327\|TPD52_HUMAN; sp\|P55327-7\|TPD52_HUMAN; sp\|P55327-6\|TPD52_HUMAN; sp\|P55327-5\|TPD52_HUMAN; sp\|P55327-4\|TPD52_HUMAN; sp\|P55327-3\|TPD52_HUMAN; sp\|P55327-2\|TPD52_HUMAN | **Uncharacterized protein OS=Homo sapiens PE=4 SV=2; Uncharacterized protein OS=Homo sapiens PE=4 SV=2; Tumor protein D52 OS=Homo sapiens GN=TPD52 PE=1 SV=2; Isoform 7 of Tumor protein D52 OS=Homo sapiens GN=TPD52; Isoform 6 of Tumor protein D52 OS=Homo sapiens GN=TPD52; Isoform 5 of Tumor protein D52 OS=Homo sapiens GN=TPD52; Isoform 4 of Tumor protein D52 OS=Homo sapiens GN=TPD52; Isoform 3 of Tumor protein D52 OS=Homo sapiens GN=TPD52; Isoform 2 of Tumor protein D52 OS=Homo sapiens GN=TPD52** | | 3 | 4 | 1.676 | 0.165 | 0.221 | 0.025 |
|  | **Peptidyl-prolyl isomerases** | | | | | | |  |  |  |
| **88** |  | sp\|P62942\|FKB1A_HUMAN | **Peptidyl-prolyl cis-trans isomerase FKBP1A OS=Homo sapiens GN=FKBP1A PE=1 SV=2** | | 6 | 7 | 2.544 | 0.401 | 0.117 | 0.030 |
| **89** |  | sp\|Q00688\|FKBP3_HUMAN | **Peptidyl-prolyl cis-trans isomerase FKBP3 OS=Homo sapiens GN=FKBP3 PE=1 SV=1** | | 3 | 4 | 1.819 | 0.543 | 0.279 | 0.025 |
|  | **coiled-coil domain-containing proteins** | | | | | | |  |  |  |
| **90** |  | sp\|Q9Y2S6\|TMA7_HUMAN | **Translation machinery-associated protein 7 OS=Homo sapiens GN=TMA7 PE=1 SV=1** | | 3 | 4 | 1.223 | -0.046 | 0.281 | 0.002 |
|  | **Misc** |  |  | |  |  |  |  |  |  |
| **91** |  | sp\|P05455\|LA_HUMAN | **Lupus La protein OS=Homo sapiens GN=SSB PE=1 SV=2** | | 3 | 4 | 1.097 | -0.492 | 0.204 | 0.022 |
| **92** |  | tr\|E7ERJ7\|E7ERJ7_HUMAN; tr\|E7EQV3\|E7EQV3_HUMAN; tr\|A0A087WTT1\|A0A087WTT1_HUMAN; sp\|P11940\|PABP1_HUMAN; sp\|P11940-2\|PABP1_HUMAN; tr\|H0YAR2\|H0YAR2_HUMAN | **Polyadenylate-binding protein 1 OS=Homo sapiens GN=PABPC1 PE=1 SV=1; Polyadenylate-binding protein 1 OS=Homo sapiens GN=PABPC1 PE=1 SV=1; Polyadenylate-binding protein 1 OS=Homo sapiens GN=PABPC1 PE=1 SV=1; Polyadenylate-binding protein 1 OS=Homo sapiens GN=PABPC1 PE=1 SV=2; Isoform 2 of Polyadenylate-binding protein 1 OS=Homo sapiens GN=PABPC1; Polyadenylate-binding protein 1 (Fragment) OS=Homo sapiens GN=PABPC1 PE=1 SV=1** | | 3 | 4 | 0.410 | -0.811 | 0.295 | 0.045 |
| **93** |  | sp\|Q9UKY7\|CDV3_HUMAN; sp\|Q9UKY7-2\|CDV3_HUMAN | **Protein CDV3 homolog OS=Homo sapiens GN=CDV3 PE=1 SV=1; Isoform 2 of Protein CDV3 homolog OS=Homo sapiens GN=CDV3** | | 3 | 4 | 1.479 | 0.467 | 0.363 | 0.041 |
| **94** |  | tr\|H0YBZ2\|H0YBZ2_HUMAN; sp\|P04233\|HG2A_HUMAN | **HLA class II histocompatibility antigen gamma chain (Fragment) OS=Homo sapiens GN=CD74 PE=1 SV=1; HLA class II histocompatibility antigen gamma chain OS=Homo sapiens GN=CD74 PE=1 SV=3** | | 3 | 4 | 2.100 | 0.359 | 0.175 | 0.001 |
| **95** |  | sp\|P12956\|XRCC6_HUMAN; sp\|P12956-2\|XRCC6_HUMAN | **X-ray repair cross-complementing protein 6 OS=Homo sapiens GN=XRCC6 PE=1 SV=2; Isoform 2 of X-ray repair cross-complementing protein 6 OS=Homo sapiens GN=XRCC6** | | 3 | 4 | 0.878 | -0.342 | 0.295 | 0.002 |
| **96** |  | sp\|P49321\|NASP_HUMAN; sp\|P49321-3\|NASP_HUMAN | **Nuclear autoantigenic sperm protein OS=Homo sapiens GN=NASP PE=1 SV=2; Isoform 3 of Nuclear autoantigenic sperm protein OS=Homo sapiens GN=NASP** | | 3 | 4 | 2.614 | 0.690 | 0.146 | 0.021 |
| **97** |  | sp\|Q9C005\|DPY30_HUMAN | **Protein dpy-30 homolog OS=Homo sapiens GN=DPY30 PE=1 SV=1** | | 3 | 4 | 0.828 | -0.638 | 0.231 | 0.044 |
| **98** |  | sp\|Q9Y265\|RUVB1_HUMAN | **RuvB-like 1 OS=Homo sapiens GN=RUVBL1 PE=1 SV=1** | | 3 | 4 | 0.242 | -0.923 | 0.312 | 0.028 |
| **99** |  | tr\|E9PRZ9\|E9PRZ9_HUMAN; tr\|E9PM92\|E9PM92_HUMAN; sp\|O00193\|SMAP_HUMAN | **Small acidic protein (Fragment) OS=Homo sapiens GN=C11orf58 PE=1 SV=1; Small acidic protein (Fragment) OS=Homo sapiens GN=C11orf58 PE=1 SV=1; Small acidic protein OS=Homo sapiens GN=SMAP PE=1 SV=1** | | 6 | 7 | 1.746 | 0.443 | 0.272 | 0.011 |
| **100** |  | sp\|Q15847\|ADIRF_HUMAN | **Adipogenesis regulatory factor OS=Homo sapiens GN=ADIRF PE=1 SV=1** | | 6 | 7 | 0.837 | -0.329 | 0.311 | 0.002 |
| **101** |  | tr\|Q5TAQ8\|Q5TAQ8_HUMAN; sp\|Q5TAQ9-2\|DCAF8_HUMAN | **DDB1- and CUL4-associated factor 8 (Fragment) OS=Homo sapiens GN=DCAF8 PE=1 SV=1; Isoform 2 of DDB1- and CUL4-associated factor 8 OS=Homo sapiens GN=DCAF8** | | 6 | 7 | 2.502 | 0.229 | 0.103 | 0.027 |
| **102** |  | tr\|H3BU16\|H3BU16_HUMAN; tr\|H3BTV5\|H3BTV5_HUMAN; tr\|H3BMV3\|H3BMV3_HUMAN; tr\|H3BMT0\|H3BMT0_HUMAN; tr\|A6NGP5\|A6NGP5_HUMAN; sp\|Q9H910\|HN1L_HUMAN; sp\|Q9H910-3\|HN1L_HUMAN; sp\|Q9H910-2\|HN1L_HUMAN | **Hematological and neurological-expressed 1-like protein (Fragment) OS=Homo sapiens GN=HN1L PE=1 SV=1; Hematological and neurological-expressed 1-like protein (Fragment) OS=Homo sapiens GN=HN1L PE=1 SV=1; Hematological and neurological-expressed 1-like protein (Fragment) OS=Homo sapiens GN=HN1L PE=1 SV=1; Hematological and neurological-expressed 1-like protein (Fragment) OS=Homo sapiens GN=HN1L PE=1 SV=1; Hematological and neurological-expressed 1-like protein OS=Homo sapiens GN=HN1L PE=2 SV=2; Hematological and neurological expressed 1-like protein OS=Homo sapiens GN=HN1L PE=1 SV=1; Isoform 3 of Hematological and neurological expressed 1-like protein OS=Homo sapiens GN=HN1L; Isoform 2 of Hematological and neurological expressed 1-like protein OS=Homo sapiens GN=HN1L** | | 3 | 4 | 1.199 | -0.084 | 0.277 | 0.006 |
| **103** |  | sp\|P33241\|LSP1_HUMAN | **Lymphocyte-specific protein 1 OS=Homo sapiens GN=LSP1 PE=1 SV=1** | | 3 | 4 | 0.725 | -0.861 | 0.205 | 0.039 |
| **104** |  | sp\|Q6GMV3\|PTRD1_HUMAN | **Putative peptidyl-tRNA hydrolase PTRHD1 OS=Homo sapiens GN=PTRHD1 PE=1 SV=1** | | 3 | 4 | 1.359 | 0.288 | 0.343 | 0.042 |

**Supplementary Table 2**: Protein abundances in CRS subgroups (based on polyposis) were compared with healthy controls. Average protein signals in each cohort were compared, and a two-tailed t-test was performed to test for significance.

|  | **Protein name** | **CRSwNP Vs Healthy** | | | | |  | **CRSsNP Vs Healthy** | | | | |
| --- | --- | --- | --- | --- | --- | --- | --- | --- | --- | --- | --- | --- |
|  |  | **# CRSwNP** | **# H** | **CRSwNP Average** | **H Average** | **p (two-tailed t-test)** |  | **#CRSsNP** | **# H** | **CRSsNP Average** | **H Average** | **p (two-tailed t-test)** |
| 1 | **Hexokinase-1 OS=Homo sapiens GN=HK1 PE=1 SV=3; Isoform 4 of Hexokinase-1 OS=Homo sapiens GN=HK1; Isoform 3 of Hexokinase-1 OS=Homo sapiens GN=HK1; Isoform 2 of Hexokinase-1 OS=Homo sapiens GN=HK1** | 2 | 3 | -0.24 | 1.43 | 0.034 |  | 2.00 | 3.00 | 0.26 | 1.43 | 0.400 |
| 2 | **Fructose-bisphosphate aldolase A OS=Homo sapiens GN=ALDOA PE=1 SV=2; Isoform 2 of Fructose-bisphosphate aldolase A OS=Homo sapiens GN=ALDOA** | 4 | 9 | -0.09 | 0.69 | 0.073 |  | 6.00 | 9.00 | 0.12 | 0.69 | 0.038 |
| 4 | **Pyruvate kinase PKM OS=Homo sapiens GN=PKM PE=1 SV=4** | 4 | 9 | -0.60 | 0.13 | 0.034 |  | 6.00 | 9.00 | -0.25 | 0.13 | 0.238 |
| 5 | **Phosphoglucomutase-1 OS=Homo sapiens GN=PGM1 PE=1 SV=3** | 2 | 3 | -0.71 | 0.01 | 0.004 |  | 2.00 | 3.00 | -1.19 | 0.01 | 0.286 |
| 6 | **6-phosphogluconolactonase OS=Homo sapiens GN=PGLS PE=1 SV=2** | 0 | 3 |  | 0.54 |  |  | 3.00 | 3.00 | -0.49 | 0.54 | 0.033 |
| 7 | **Hydroxymethylglutaryl-CoA synthase, cytoplasmic OS=Homo sapiens GN=HMGCS1 PE=1 SV=2** | 2 | 3 | -0.63 | 0.66 | 0.057 |  | 2.00 | 3.00 | -0.89 | 0.66 | 0.036 |
| 8 | **Argininosuccinate lyase OS=Homo sapiens GN=ASL PE=1 SV=4; Isoform 3 of Argininosuccinate lyase OS=Homo sapiens GN=ASL; Isoform 2 of Argininosuccinate lyase OS=Homo sapiens GN=ASL** | 2 | 3 | -1.32 | 0.92 | 0.106 |  | 2.00 | 3.00 | -0.69 | 0.92 | 0.058 |
| 9 | **2,4-dienoyl-CoA reductase, mitochondrial OS=Homo sapiens GN=DECR1 PE=1 SV=1; Isoform 2 of 2,4-dienoyl-CoA reductase, mitochondrial OS=Homo sapiens GN=DECR1** | 2 | 3 | 1.58 | 4.62 | 0.053 |  | 2.00 | 3.00 | -0.89 | 4.62 | 0.265 |
| 10 | **Prosaposin OS=Homo sapiens GN=PSAP PE=1 SV=2; Saposin-D OS=Homo sapiens GN=PSAP PE=1 SV=1; Prosaposin OS=Homo sapiens GN=PSAP PE=1 SV=2; Isoform Sap-mu-9 of Prosaposin OS=Homo sapiens GN=PSAP; Isoform Sap-mu-6 of Prosaposin OS=Homo sapiens GN=PSAP** | 4 | 6 | -0.44 | 0.30 | 0.067 |  | 3.00 | 6.00 | 0.02 | 0.30 | 0.410 |
| 11 | **Acetyl-CoA acetyltransferase, cytosolic OS=Homo sapiens GN=ACAT2 PE=1 SV=2; Isoform 2 of Acetyl-CoA acetyltransferase, cytosolic OS=Homo sapiens GN=ACAT2** | 0 | 3 |  | 2.00 |  |  | 3.00 | 3.00 | 0.67 | 2.00 | 0.048 |
| 12 | **Histidine triad nucleotide-binding protein 2, mitochondrial OS=Homo sapiens GN=HINT2 PE=1 SV=1** | 2 | 3 | -0.13 | 1.41 | 0.016 |  | 2.00 | 3.00 | 0.27 | 1.41 | 0.032 |
| 13 | **Aldo-keto reductase family 1 member C1 OS=Homo sapiens GN=AKR1C1 PE=4 SV=1; Aldo-keto reductase family 1 member C1 OS=Homo sapiens GN=AKR1C1 PE=1 SV=1** | 2 | 6 | -1.30 | 0.05 | 0.003 |  | 5.00 | 6.00 | -0.71 | 0.05 | 0.111 |
| 14 | **Aldo-keto reductase family 1 member C3 OS=Homo sapiens GN=AKR1C3 PE=4 SV=1; Aldo-keto reductase family 1 member C3 OS=Homo sapiens GN=AKR1C3 PE=1 SV=4** | 2 | 6 | -0.69 | 0.27 | 0.160 |  | 5.00 | 6.00 | -0.93 | 0.27 | 0.030 |
| 15 | **Proline synthase co-transcribed bacterial homolog protein (Fragment) OS=Homo sapiens GN=PROSC PE=1 SV=1; Proline synthase co-transcribed bacterial homolog protein OS=Homo sapiens GN=PROSC PE=1 SV=1** | 2 | 6 | 0.09 | 0.95 | 0.113 |  | 5.00 | 6.00 | -0.89 | 0.95 | 0.017 |
| 16 | **Malate dehydrogenase, mitochondrial OS=Homo sapiens GN=MDH2 PE=1 SV=3** | 4 | 9 | -0.16 | 0.84 | 0.007 |  | 6.00 | 9.00 | 0.28 | 0.84 | 0.143 |
| 17 | **Stress-70 protein, mitochondrial OS=Homo sapiens GN=HSPA9 PE=1 SV=2** | 2 | 3 | -0.20 | 1.56 | 0.127 |  | 2.00 | 3.00 | 0.27 | 1.56 | 0.143 |
| 18 | **ATP synthase subunit alpha, mitochondrial OS=Homo sapiens GN=ATP5A1 PE=1 SV=1; Isoform 2 of ATP synthase subunit alpha, mitochondrial OS=Homo sapiens GN=ATP5A1** | 2 | 3 | -0.35 | 2.52 | 0.021 |  | 2.00 | 3.00 | -0.20 | 2.52 | 0.024 |
| 19 | **Creatine kinase B-type OS=Homo sapiens GN=CKB PE=1 SV=1** | 4 | 9 | -0.08 | 0.86 | 0.191 |  | 6.00 | 9.00 | -0.46 | 0.86 | 0.006 |
| 20 | **Adenylosuccinate synthetase isozyme 2 OS=Homo sapiens GN=ADSS PE=1 SV=3** | 2 | 3 | 0.32 | 3.33 | 0.018 |  | 2.00 | 3.00 | 1.29 | 3.33 | 0.341 |
| 21 | **Adenylate kinase 2, mitochondrial OS=Homo sapiens GN=AK2 PE=1 SV=1; Adenylate kinase 2, mitochondrial OS=Homo sapiens GN=AK2 PE=1 SV=2; Isoform 2 of Adenylate kinase 2, mitochondrial OS=Homo sapiens GN=AK2; Isoform 5 of Adenylate kinase 2, mitochondrial OS=Homo sapiens GN=AK2** | 2 | 3 | 0.88 | 2.72 | 0.005 |  | 2.00 | 3.00 | 1.39 | 2.72 | 0.192 |
| 22 | **dCTP pyrophosphatase 1 OS=Homo sapiens GN=DCTPP1 PE=1 SV=1** | 2 | 3 | -0.71 | 1.41 | 0.169 |  | 2.00 | 3.00 | -0.54 | 1.41 | 0.138 |
| 23 | **GMP reductase OS=Homo sapiens GN=GMPR2 PE=3 SV=1; GMP reductase 2 OS=Homo sapiens GN=GMPR2 PE=1 SV=1; GMP reductase 2 OS=Homo sapiens GN=GMPR2 PE=1 SV=1; GMP reductase OS=Homo sapiens GN=GMPR2 PE=1 SV=1; GMP reductase OS=Homo sapiens GN=GMPR2 PE=1 SV=1; GMP reductase 2 OS=Homo sapiens GN=GMPR2 PE=1 SV=1; Isoform 2 of GMP reductase 2 OS=Homo sapiens GN=GMPR2** | 2 | 6 | -0.17 | 0.88 | 0.221 |  | 5.00 | 6.00 | -0.33 | 0.88 | 0.044 |
| 24 | **Porphobilinogen deaminase OS=Homo sapiens GN=HMBS PE=1 SV=1; Porphobilinogen deaminase OS=Homo sapiens GN=HMBS PE=1 SV=2; Isoform 4 of Porphobilinogen deaminase OS=Homo sapiens GN=HMBS; Isoform 3 of Porphobilinogen deaminase OS=Homo sapiens GN=HMBS; Isoform 2 of Porphobilinogen deaminase OS=Homo sapiens GN=HMBS** | 2 | 3 | -0.26 | 1.30 | 0.057 |  | 2.00 | 3.00 | 0.37 | 1.30 | 0.171 |
| 25 | **Sialic acid synthase OS=Homo sapiens GN=NANS PE=1 SV=2** | 2 | 6 | -0.62 | 0.42 | 0.010 |  | 5.00 | 6.00 | -0.49 | 0.42 | 0.086 |
| 26 | **Aldehyde dehydrogenase, dimeric NADP-preferring OS=Homo sapiens GN=ALDH3A1 PE=1 SV=3** | 4 | 9 | -1.06 | -0.11 | 0.228 |  | 6.00 | 9.00 | -1.01 | -0.11 | 0.038 |
| 27 | **Alcohol dehydrogenase 1C OS=Homo sapiens GN=ADH1C PE=1 SV=2** | 4 | 9 | -1.39 | 0.05 | 0.016 |  | 6.00 | 9.00 | -0.56 | 0.05 | 0.144 |
| 28 | **Aldo-keto reductase family 1 member B10 OS=Homo sapiens GN=AKR1B10 PE=1 SV=2** | 2 | 3 | -1.29 | 0.24 | 0.060 |  | 2.00 | 3.00 | -1.18 | 0.24 | 0.018 |
| 29 | **Isocitrate dehydrogenase [NADP] cytoplasmic OS=Homo sapiens GN=IDH1 PE=1 SV=2** | 4 | 9 | 0.12 | 1.33 | 0.097 |  | 6.00 | 9.00 | 0.22 | 1.33 | 0.007 |
| 30 | **Sorbitol dehydrogenase OS=Homo sapiens GN=SORD PE=1 SV=1; Sorbitol dehydrogenase OS=Homo sapiens GN=SORD PE=1 SV=4** | 4 | 9 | -0.50 | 0.38 | 0.137 |  | 6.00 | 9.00 | -0.70 | 0.38 | 0.066 |
| 31 | **Quinone oxidoreductase (Fragment) OS=Homo sapiens GN=CRYZ PE=1 SV=1; Quinone oxidoreductase (Fragment) OS=Homo sapiens GN=CRYZ PE=1 SV=1; Quinone oxidoreductase OS=Homo sapiens GN=CRYZ PE=1 SV=1; Isoform 3 of Quinone oxidoreductase OS=Homo sapiens GN=CRYZ** | 2 | 3 | -0.36 | 1.73 | 0.031 |  | 2.00 | 3.00 | -0.97 | 1.73 | 0.161 |
| 32 | **Tight junction protein ZO-2 OS=Homo sapiens GN=TJP2 PE=1 SV=2; Isoform 7 of Tight junction protein ZO-2 OS=Homo sapiens GN=TJP2; Isoform 6 of Tight junction protein ZO-2 OS=Homo sapiens GN=TJP2; Isoform C1 of Tight junction protein ZO-2 OS=Homo sapiens GN=TJP2** | 2 | 3 | -0.44 | 1.25 | 0.006 |  | 2.00 | 3.00 | -0.48 | 1.25 | 0.005 |
| 33 | **Prothymosin alpha (Fragment) OS=Homo sapiens GN=PTMA PE=1 SV=1; Prothymosin alpha OS=Homo sapiens GN=PTMA PE=1 SV=1; Prothymosin alpha OS=Homo sapiens GN=PTMA PE=1 SV=1; Prothymosin alpha OS=Homo sapiens GN=PTMA PE=1 SV=1; Prothymosin alpha OS=Homo sapiens GN=PTMA PE=1 SV=2; Isoform 2 of Prothymosin alpha OS=Homo sapiens GN=PTMA** | 4 | 9 | 0.41 | 1.34 | 0.155 |  | 6.00 | 9.00 | 0.67 | 1.34 | 0.168 |
| 34 | **Granulins OS=Homo sapiens GN=GRN PE=1 SV=2; Isoform 2 of Granulins OS=Homo sapiens GN=GRN** | 2 | 3 | 0.33 | 2.34 | 0.163 |  | 2.00 | 3.00 | 1.06 | 2.34 | 0.114 |
| 35 | **Growth-regulated alpha protein OS=Homo sapiens GN=CXCL1 PE=1 SV=1** | 2 | 3 | 0.90 | 2.52 | 0.217 |  | 2.00 | 3.00 | 0.50 | 2.52 | 0.115 |
| 36 | **Clusterin OS=Homo sapiens GN=CLU PE=1 SV=1; Isoform 5 of Clusterin OS=Homo sapiens GN=CLU; Isoform 4 of Clusterin OS=Homo sapiens GN=CLU; Isoform 2 of Clusterin OS=Homo sapiens GN=CLU** | 4 | 6 | 0.59 | 1.41 | 0.013 |  | 3.00 | 6.00 | 0.48 | 1.41 | 0.264 |
| 37 | **Na(+)/H(+) exchange regulatory cofactor NHE-RF1 OS=Homo sapiens GN=SLC9A3R1 PE=1 SV=4** | 4 | 6 | 0.64 | 1.83 | 0.035 |  | 3.00 | 6.00 | 1.05 | 1.83 | 0.034 |
| 38 | **Ezrin OS=Homo sapiens GN=EZR PE=1 SV=3; Ezrin OS=Homo sapiens GN=EZR PE=1 SV=4** | 4 | 9 | -0.11 | 0.91 | 0.039 |  | 6.00 | 9.00 | 0.09 | 0.91 | 0.045 |
| 39 | **Tropomodulin-3 OS=Homo sapiens GN=TMOD3 PE=1 SV=1** | 2 | 3 | -0.18 | 0.93 | 0.016 |  | 2.00 | 3.00 | -0.10 | 0.93 | 0.045 |
| 40 | **Nucleobindin-1 OS=Homo sapiens GN=NUCB1 PE=1 SV=4** | 2 | 6 | 0.26 | 1.87 | 0.011 |  | 5.00 | 6.00 | 0.88 | 1.87 | 0.075 |
| 41 | **Epididymis secretory protein Li 109 OS=Homo sapiens GN=HEL-S-109 PE=2 SV=1** | 2 | 6 | -0.51 | 0.37 | 0.041 |  | 5.00 | 6.00 | -0.31 | 0.37 | 0.084 |
| 42 | **Histidine triad nucleotide-binding protein 1 OS=Homo sapiens GN=HINT1 PE=1 SV=2** | 4 | 9 | 0.21 | 1.31 | 0.006 |  | 6.00 | 9.00 | 0.52 | 1.31 | 0.067 |
| 43 | **Isoform 2 of Glyoxalase domain-containing protein 4 OS=Homo sapiens GN=GLOD4** | 4 | 9 | -0.27 | 0.77 | 0.051 |  | 6.00 | 9.00 | 0.06 | 0.77 | 0.047 |
| 44 | **Lactoylglutathione lyase OS=Homo sapiens GN=GLO1 PE=1 SV=4** | 2 | 6 | -0.39 | 1.99 | 0.047 |  | 5.00 | 6.00 | -0.03 | 1.99 | 0.058 |
| 45 | **Thioredoxin domain-containing protein 12 OS=Homo sapiens GN=TXNDC12 PE=1 SV=1** | 2 | 3 | 4.32 | 6.75 | 0.004 |  | 2.00 | 3.00 | 3.02 | 6.75 | 0.320 |
| 46 | **Liver carboxylesterase 1 OS=Homo sapiens GN=CES1 PE=1 SV=2; Isoform 3 of Liver carboxylesterase 1 OS=Homo sapiens GN=CES1; Isoform 2 of Liver carboxylesterase 1 OS=Homo sapiens GN=CES1** | 4 | 9 | -0.72 | 0.71 | 0.001 |  | 5.00 | 9.00 | -0.89 | 0.71 | 0.061 |
| 47 | **Glutathione S-transferase omega-1 OS=Homo sapiens GN=GSTO1 PE=1 SV=2** | 2 | 6 | -0.46 | 0.24 | 0.025 |  | 5.00 | 6.00 | -0.61 | 0.24 | 0.018 |
| 48 | **T-complex protein 1 subunit eta OS=Homo sapiens GN=CCT7 PE=1 SV=2** | 2 | 3 | -0.58 | 1.01 | 0.053 |  | 2.00 | 3.00 | -0.71 | 1.01 | 0.038 |
| 49 | **60 kDa heat shock protein, mitochondrial OS=Homo sapiens GN=HSPD1 PE=1 SV=2** | 4 | 6 | 0.25 | 1.27 | 0.060 |  | 3.00 | 6.00 | 0.78 | 1.27 | 0.338 |
| 50 | **Heat shock protein beta-1 OS=Homo sapiens GN=HSPB1 PE=1 SV=2** | 4 | 9 | 0.25 | 1.33 | 0.177 |  | 6.00 | 9.00 | 0.64 | 1.33 | 0.066 |
| 51 | **Heat shock protein HSP 90-beta OS=Homo sapiens GN=HSP90AB1 PE=1 SV=4** | 2 | 6 | -1.08 | 0.32 | 0.183 |  | 5.00 | 6.00 | -0.33 | 0.32 | 0.086 |
| 52 | **Hsc70-interacting protein OS=Homo sapiens GN=ST13 PE=1 SV=2** | 2 | 6 | -0.51 | 0.86 | 0.128 |  | 5.00 | 6.00 | 0.03 | 0.86 | 0.086 |
| 53 | **Endoplasmin OS=Homo sapiens GN=HSP90B1 PE=1 SV=1** | 4 | 6 | 0.52 | 1.60 | 0.112 |  | 3.00 | 6.00 | 0.33 | 1.60 | 0.034 |
| 54 | **Protein disulfide-isomerase A6 OS=Homo sapiens GN=PDIA6 PE=1 SV=1; Isoform 5 of Protein disulfide-isomerase A6 OS=Homo sapiens GN=PDIA6; Isoform 4 of Protein disulfide-isomerase A6 OS=Homo sapiens GN=PDIA6; Isoform 3 of Protein disulfide-isomerase A6 OS=Homo sapiens GN=PDIA6; Isoform 2 of Protein disulfide-isomerase A6 OS=Homo sapiens GN=PDIA6** | 2 | 3 | -0.47 | 1.01 | 0.002 |  | 2.00 | 3.00 | 0.03 | 1.01 | 0.007 |
| 55 | **Nuclear ubiquitous casein and cyclin-dependent kinase substrate 1 OS=Homo sapiens GN=NUCKS1 PE=1 SV=1; Isoform 2 of Nuclear ubiquitous casein and cyclin-dependent kinase substrate 1 OS=Homo sapiens GN=NUCKS1** | 2 | 3 | 0.51 | 2.78 | 0.077 |  | 2.00 | 3.00 | 1.46 | 2.78 | 0.009 |
| 56 | **Protein phosphatase 1A OS=Homo sapiens GN=PPM1A PE=1 SV=1; Isoform 3 of Protein phosphatase 1A OS=Homo sapiens GN=PPM1A** | 2 | 3 | -0.59 | 1.43 | 0.017 |  | 2.00 | 3.00 | -2.67 | 1.43 | 0.262 |
| 57 | **Chloride intracellular channel protein 1 OS=Homo sapiens GN=CLIC1 PE=1 SV=4** | 2 | 6 | -0.82 | -0.09 | 0.383 |  | 5.00 | 6.00 | -0.93 | -0.09 | 0.028 |
| 58 | **Metallothionein-1M OS=Homo sapiens GN=MT1M PE=2 SV=2; Metallothionein-1X OS=Homo sapiens GN=MT1X PE=1 SV=1; Metallothionein-1G OS=Homo sapiens GN=MT1G PE=1 SV=2; Isoform 2 of Metallothionein-1G OS=Homo sapiens GN=MT1G; Metallothionein-1E OS=Homo sapiens GN=MT1E PE=1 SV=1; Metallothionein-2 OS=Homo sapiens GN=MT2A PE=1 SV=1** | 2 | 3 | 0.05 | 0.97 | 0.003 |  | 2.00 | 3.00 | 0.12 | 0.97 | 0.085 |
| 59 | **Neutral alpha-glucosidase AB OS=Homo sapiens GN=GANAB PE=1 SV=3; Isoform 2 of Neutral alpha-glucosidase AB OS=Homo sapiens GN=GANAB** | 2 | 3 | -0.31 | 0.57 | 0.020 |  | 2.00 | 3.00 | -0.47 | 0.57 | 0.022 |
| 60 | **Small ubiquitin-related modifier 2 OS=Homo sapiens GN=SUMO2 PE=1 SV=3; Isoform 2 of Small ubiquitin-related modifier 2 OS=Homo sapiens GN=SUMO2** | 4 | 6 | -0.11 | 0.91 | 0.019 |  | 3.00 | 6.00 | 0.04 | 0.91 | 0.178 |
| 61 | **Ubiquitin-conjugating enzyme E2 L3 OS=Homo sapiens GN=UBE2L3 PE=1 SV=1; Isoform 3 of Ubiquitin-conjugating enzyme E2 L3 OS=Homo sapiens GN=UBE2L3; Isoform 2 of Ubiquitin-conjugating enzyme E2 L3 OS=Homo sapiens GN=UBE2L3** | 2 | 6 | 0.20 | 1.77 | 0.068 |  | 5.00 | 6.00 | 0.88 | 1.77 | 0.074 |
| 62 | **Proteasome subunit alpha type OS=Homo sapiens GN=PSMA6 PE=1 SV=1; Proteasome subunit alpha type-6 OS=Homo sapiens GN=PSMA6 PE=1 SV=1; Isoform 2 of Proteasome subunit alpha type-6 OS=Homo sapiens GN=PSMA6** | 2 | 6 | 0.41 | 1.09 | 0.454 |  | 5.00 | 6.00 | 0.34 | 1.09 | 0.041 |
| 63 | **Proteasome subunit alpha type-7 OS=Homo sapiens GN=PSMA7 PE=1 SV=1** | 4 | 9 | -1.90 | 1.09 | 0.175 |  | 6.00 | 9.00 | -0.04 | 1.09 | 0.096 |
| 64 | **Proteasome subunit beta type-10 OS=Homo sapiens GN=PSMB10 PE=1 SV=1** | 2 | 3 | -0.34 | 0.58 | 0.305 |  | 2.00 | 3.00 | -0.65 | 0.58 | 0.071 |
| 65 | **Angiotensinogen OS=Homo sapiens GN=AGT PE=1 SV=1** | 2 | 6 | 1.35 | -1.88 | 0.010 |  | 5.00 | 6.00 | -0.16 | -1.88 | 0.093 |
| 66 | **Calpastatin OS=Homo sapiens GN=CAST PE=1 SV=1; Calpastatin OS=Homo sapiens GN=CAST PE=1 SV=1; Calpastatin (Fragment) OS=Homo sapiens GN=CAST PE=1 SV=1; Calpastatin OS=Homo sapiens GN=CAST PE=2 SV=1; Calpastatin OS=Homo sapiens GN=CAST PE=1 SV=4; Isoform 8 of Calpastatin OS=Homo sapiens GN=CAST; Isoform 6 of Calpastatin OS=Homo sapiens GN=CAST; Isoform 5 of Calpastatin OS=Homo sapiens GN=CAST; Isoform 2 of Calpastatin OS=Homo sapiens GN=CAST; Isoform 10 of Calpastatin OS=Homo sapiens GN=CAST** | 2 | 6 | -0.35 | 0.69 | 0.082 |  | 5.00 | 6.00 | 0.12 | 0.69 | 0.103 |
| 67 | **Elafin OS=Homo sapiens GN=PI3 PE=1 SV=3** | 4 | 9 | -0.12 | 0.57 | 0.296 |  | 6.00 | 9.00 | -0.72 | 0.57 | 0.016 |
| 68 | **THO complex subunit 4 OS=Homo sapiens GN=ALYREF PE=1 SV=1; THO complex subunit 4 OS=Homo sapiens GN=ALYREF PE=1 SV=3** | 2 | 3 | -0.07 | 2.92 | 0.004 |  | 2.00 | 3.00 | 0.99 | 2.92 | 0.030 |
| 69 | **Clathrin light chain A OS=Homo sapiens GN=CLTA PE=1 SV=1; Isoform 4 of Clathrin light chain A OS=Homo sapiens GN=CLTA; Isoform 3 of Clathrin light chain A OS=Homo sapiens GN=CLTA; Isoform Non-brain of Clathrin light chain A OS=Homo sapiens GN=CLTA** | 2 | 3 | -0.57 | 0.47 | 0.007 |  | 2.00 | 3.00 | 0.12 | 0.47 | 0.100 |
| 70 | **Beta-arrestin-1 OS=Homo sapiens GN=ARRB1 PE=1 SV=2; Isoform 1B of Beta-arrestin-1 OS=Homo sapiens GN=ARRB1** | 2 | 3 | -0.66 | 1.15 | 0.024 |  | 2.00 | 3.00 | -0.13 | 1.15 | 0.018 |
| 71 | **Ras-related C3 botulinum toxin substrate 1 OS=Homo sapiens GN=RAC1 PE=1 SV=1; Isoform B of Ras-related C3 botulinum toxin substrate 1 OS=Homo sapiens GN=RAC1** | 2 | 6 | -0.63 | 1.09 | 0.033 |  | 5.00 | 6.00 | -0.07 | 1.09 | 0.028 |
| 72 | **Ras-related protein Rab-1B OS=Homo sapiens GN=RAB1B PE=1 SV=1** | 2 | 3 | -0.70 | 0.06 | 0.151 |  | 2.00 | 3.00 | -1.51 | 0.06 | 0.137 |
| 73 | **Ras-related protein Rab-14 (Fragment) OS=Homo sapiens GN=RAB14 PE=1 SV=1; Ras-related protein Rab-14 OS=Homo sapiens GN=RAB14 PE=1 SV=4** | 2 | 3 | -0.37 | -3.47 | 0.054 |  | 2.00 | 3.00 | -0.32 | -3.47 | 0.022 |
| 74 | **Ran-specific GTPase-activating protein OS=Homo sapiens GN=RANBP1 PE=1 SV=1; Ran-specific GTPase-activating protein (Fragment) OS=Homo sapiens GN=RANBP1 PE=1 SV=2; Ran-specific GTPase-activating protein OS=Homo sapiens GN=RANBP1 PE=1 SV=1; Isoform 2 of Ran-specific GTPase-activating protein OS=Homo sapiens GN=RANBP1** | 2 | 3 | -0.57 | 2.01 | 0.020 |  | 2.00 | 3.00 | 0.14 | 2.01 | 0.048 |
| 75 | **Calcyphosin-like protein OS=Homo sapiens GN=CAPSL PE=4 SV=1; Calcyphosin-like protein (Fragment) OS=Homo sapiens GN=CAPSL PE=4 SV=1; Calcyphosin-like protein OS=Homo sapiens GN=CAPSL PE=2 SV=4** | 0 | 3 |  | 2.05 |  |  | 3.00 | 3.00 | 0.64 | 2.05 | 0.017 |
| 76 | **Histone H2A.V OS=Homo sapiens GN=H2AFV PE=1 SV=3; Histone H2A.Z OS=Homo sapiens GN=H2AFZ PE=1 SV=2** | 2 | 3 | 0.12 | 2.53 | 0.134 |  | 2.00 | 3.00 | -0.01 | 2.53 | 0.008 |
| 77 | **High mobility group protein B1 OS=Homo sapiens GN=HMGB1 PE=1 SV=3** | 4 | 9 | 0.09 | 0.71 | 0.047 |  | 6.00 | 9.00 | 0.29 | 0.71 | 0.116 |
| 78 | **PC4 and SFRS1-interacting protein OS=Homo sapiens GN=PSIP1 PE=1 SV=1** | 2 | 3 | 1.36 | 2.69 | 0.039 |  | 2.00 | 3.00 | 1.05 | 2.69 | 0.006 |
| 79 | **40S ribosomal protein S11 OS=Homo sapiens GN=RPS11 PE=1 SV=3** | 2 | 3 | 0.17 | 1.90 | 0.113 |  | 2.00 | 3.00 | 0.20 | 1.90 | 0.154 |
| 80 | **40S ribosomal protein S12 OS=Homo sapiens GN=RPS12 PE=1 SV=3** | 2 | 3 | 0.45 | -2.81 | 0.008 |  | 2.00 | 3.00 | 1.02 | -2.81 | 0.120 |
| 81 | **60S ribosomal protein L21 OS=Homo sapiens GN=RPL21 PE=1 SV=2** | 2 | 3 | 0.00 | 1.87 | 0.013 |  | 2.00 | 3.00 | 0.96 | 1.87 | 0.244 |
| 82 | **60S ribosomal protein L6 OS=Homo sapiens GN=RPL6 PE=1 SV=3** | 4 | 6 | 0.61 | 1.81 | 0.088 |  | 3.00 | 6.00 | -0.69 | 1.81 | 0.092 |
| 83 | **Eukaryotic translation initiation factor 1A, X-chromosomal OS=Homo sapiens GN=EIF1AX PE=1 SV=2; Eukaryotic translation initiation factor 1A, Y-chromosomal OS=Homo sapiens GN=EIF1AY PE=1 SV=4** | 2 | 3 | 0.12 | 1.61 | 0.049 |  | 2.00 | 3.00 | 0.64 | 1.61 | 0.198 |
| 84 | **Eukaryotic translation initiation factor 4H OS=Homo sapiens GN=EIF4H PE=1 SV=5; Isoform Short of Eukaryotic translation initiation factor 4H OS=Homo sapiens GN=EIF4H** | 2 | 3 | -0.32 | 1.06 | 0.024 |  | 2.00 | 3.00 | -0.31 | 1.06 | 0.114 |
| 85 | **Isoform 2 of Eukaryotic translation initiation factor 5A-1 OS=Homo sapiens GN=EIF5A** | 4 | 9 | -0.67 | -0.06 | 0.183 |  | 6.00 | 9.00 | -0.88 | -0.06 | 0.008 |
| 86 | **Heterogeneous nuclear ribonucleoprotein H OS=Homo sapiens GN=HNRNPH1 PE=1 SV=1; Heterogeneous nuclear ribonucleoprotein H OS=Homo sapiens GN=HNRNPH1 PE=1 SV=1; Heterogeneous nuclear ribonucleoprotein H2 OS=Homo sapiens GN=HNRNPH2 PE=1 SV=1; Heterogeneous nuclear ribonucleoprotein H OS=Homo sapiens GN=HNRNPH1 PE=1 SV=4** | 2 | 3 | -0.66 | 0.59 | 0.176 |  | 2.00 | 3.00 | -0.27 | 0.59 | 0.110 |
| 87 | **Splicing factor, proline- and glutamine-rich OS=Homo sapiens GN=SFPQ PE=1 SV=2; Isoform Short of Splicing factor, proline- and glutamine-rich OS=Homo sapiens GN=SFPQ** | 2 | 3 | 0.00 | 1.41 | 0.025 |  | 2.00 | 3.00 | 0.40 | 1.41 | 0.128 |
| 88 | **Isoform 2 of Elongation factor 1-gamma OS=Homo sapiens GN=EEF1G** | 4 | 9 | -0.19 | 0.61 | 0.124 |  | 6.00 | 9.00 | -0.18 | 0.61 | 0.064 |
| 89 | **Elongation factor 2 OS=Homo sapiens GN=EEF2 PE=1 SV=4** | 4 | 9 | -0.14 | 0.65 | 0.057 |  | 6.00 | 9.00 | -0.79 | 0.65 | 0.005 |
| 90 | **Uncharacterized protein OS=Homo sapiens PE=4 SV=2; Uncharacterized protein OS=Homo sapiens PE=4 SV=2; Tumor protein D52 OS=Homo sapiens GN=TPD52 PE=1 SV=2; Isoform 7 of Tumor protein D52 OS=Homo sapiens GN=TPD52; Isoform 6 of Tumor protein D52 OS=Homo sapiens GN=TPD52; Isoform 5 of Tumor protein D52 OS=Homo sapiens GN=TPD52; Isoform 4 of Tumor protein D52 OS=Homo sapiens GN=TPD52; Isoform 3 of Tumor protein D52 OS=Homo sapiens GN=TPD52; Isoform 2 of Tumor protein D52 OS=Homo sapiens GN=TPD52** | 2 | 3 | -0.39 | 1.68 | 0.011 |  | 2.00 | 3.00 | 0.72 | 1.68 | 0.208 |
| 91 | **Protein S100-A9 OS=Homo sapiens GN=S100A9 PE=1 SV=1** | 4 | 9 | 0.46 | -0.50 | 0.013 |  | 6.00 | 9.00 | 0.16 | -0.50 | 0.172 |
| 92 | **Peptidyl-prolyl cis-trans isomerase FKBP1A OS=Homo sapiens GN=FKBP1A PE=1 SV=2** | 4 | 6 | 0.52 | 2.54 | 0.078 |  | 3.00 | 6.00 | 0.24 | 2.54 | 0.084 |
| 93 | **Peptidyl-prolyl cis-trans isomerase FKBP3 OS=Homo sapiens GN=FKBP3 PE=1 SV=1** | 2 | 3 | 0.79 | 1.82 | 0.065 |  | 2.00 | 3.00 | 0.29 | 1.82 | 0.020 |
| 94 | **Translation machinery-associated protein 7 OS=Homo sapiens GN=TMA7 PE=1 SV=1** | 2 | 3 | -0.17 | 1.22 | 0.001 |  | 2.00 | 3.00 | 0.08 | 1.22 | 0.197 |
| 95 | **Lupus La protein OS=Homo sapiens GN=SSB PE=1 SV=2** | 2 | 3 | -0.74 | 1.10 | 0.108 |  | 2.00 | 3.00 | -0.24 | 1.10 | 0.048 |
| 96 | **Cell division control protein 42 homolog OS=Homo sapiens GN=CDC42 PE=1 SV=2; Isoform 1 of Cell division control protein 42 homolog OS=Homo sapiens GN=CDC42** | 2 | 3 | 0.28 | -0.33 | 0.022 |  | 2.00 | 3.00 | 0.11 | -0.33 | 0.434 |
| 97 | **Polyadenylate-binding protein 1 OS=Homo sapiens GN=PABPC1 PE=1 SV=1; Polyadenylate-binding protein 1 OS=Homo sapiens GN=PABPC1 PE=1 SV=1; Polyadenylate-binding protein 1 OS=Homo sapiens GN=PABPC1 PE=1 SV=1; Polyadenylate-binding protein 1 OS=Homo sapiens GN=PABPC1 PE=1 SV=2; Isoform 2 of Polyadenylate-binding protein 1 OS=Homo sapiens GN=PABPC1; Polyadenylate-binding protein 1 (Fragment) OS=Homo sapiens GN=PABPC1 PE=1 SV=1** | 2 | 3 | -0.77 | 0.41 | 0.051 |  | 2.00 | 3.00 | -0.85 | 0.41 | 0.049 |
| 98 | **Protein CDV3 homolog OS=Homo sapiens GN=CDV3 PE=1 SV=1; Isoform 2 of Protein CDV3 homolog OS=Homo sapiens GN=CDV3** | 2 | 3 | 0.17 | 1.48 | 0.040 |  | 2.00 | 3.00 | 0.77 | 1.48 | 0.108 |
| 99 | **HLA class II histocompatibility antigen gamma chain (Fragment) OS=Homo sapiens GN=CD74 PE=1 SV=1; HLA class II histocompatibility antigen gamma chain OS=Homo sapiens GN=CD74 PE=1 SV=3** | 2 | 3 | 0.22 | 2.10 | 0.087 |  | 2.00 | 3.00 | 0.50 | 2.10 | 0.064 |
| 100 | **X-ray repair cross-complementing protein 6 OS=Homo sapiens GN=XRCC6 PE=1 SV=2; Isoform 2 of X-ray repair cross-complementing protein 6 OS=Homo sapiens GN=XRCC6** | 2 | 3 | -0.57 | 0.88 | 0.004 |  | 2.00 | 3.00 | -0.11 | 0.88 | 0.015 |
| 101 | **Low affinity immunoglobulin gamma Fc region receptor III-B OS=Homo sapiens GN=FCGR3B PE=4 SV=1; Low affinity immunoglobulin gamma Fc region receptor III-A OS=Homo sapiens GN=FCGR3A PE=4 SV=1; Low affinity immunoglobulin gamma Fc region receptor III-A (Fragment) OS=Homo sapiens GN=FCGR3A PE=4 SV=1; Low affinity immunoglobulin gamma Fc region receptor III-B (Fragment) OS=Homo sapiens GN=FCGR3B PE=4 SV=1; Low affinity immunoglobulin gamma Fc region receptor III-B OS=Homo sapiens GN=FCGR3B PE=4 SV=1; Low affinity immunoglobulin gamma Fc region receptor III-A OS=Homo sapiens GN=FCGR3A PE=1 SV=2; Low affinity immunoglobulin gamma Fc region receptor III-B OS=Homo sapiens GN=FCGR3B PE=1 SV=2** | 2 | 6 | 1.19 | -2.29 | 0.136 |  | 5.00 | 6.00 | 0.13 | -2.29 | 0.013 |
| 102 | **Nuclear autoantigenic sperm protein OS=Homo sapiens GN=NASP PE=1 SV=2; Isoform 3 of Nuclear autoantigenic sperm protein OS=Homo sapiens GN=NASP** | 2 | 3 | 0.93 | 2.61 | 0.044 |  | 2.00 | 3.00 | 0.45 | 2.61 | 0.016 |
| 103 | **Protein dpy-30 homolog OS=Homo sapiens GN=DPY30 PE=1 SV=1** | 2 | 3 | -0.72 | 0.83 | 0.060 |  | 2.00 | 3.00 | -0.56 | 0.83 | 0.158 |
| 104 | **RuvB-like 1 OS=Homo sapiens GN=RUVBL1 PE=1 SV=1** | 2 | 3 | -1.20 | 0.24 | 0.236 |  | 2.00 | 3.00 | -0.65 | 0.24 | 0.058 |
| 105 | **Small acidic protein (Fragment) OS=Homo sapiens GN=C11orf58 PE=1 SV=1; Small acidic protein (Fragment) OS=Homo sapiens GN=C11orf58 PE=1 SV=1; Small acidic protein OS=Homo sapiens GN=SMAP PE=1 SV=1** | 4 | 6 | 0.38 | 1.75 | 0.027 |  | 3.00 | 6.00 | 0.53 | 1.75 | 0.190 |
| 106 | **Adipogenesis regulatory factor OS=Homo sapiens GN=ADIRF PE=1 SV=1** | 2 | 6 | -0.62 | 0.84 | 0.060 |  | 5.00 | 6.00 | -0.22 | 0.84 | 0.024 |
| 107 | **DDB1- and CUL4-associated factor 8 (Fragment) OS=Homo sapiens GN=DCAF8 PE=1 SV=1; Isoform 2 of DDB1- and CUL4-associated factor 8 OS=Homo sapiens GN=DCAF8** | 2 | 6 | 0.23 | 2.50 | 0.029 |  | 5.00 | 6.00 | 0.23 | 2.50 | 0.032 |
| 108 | **Hematological and neurological-expressed 1-like protein (Fragment) OS=Homo sapiens GN=HN1L PE=1 SV=1; Hematological and neurological-expressed 1-like protein (Fragment) OS=Homo sapiens GN=HN1L PE=1 SV=1; Hematological and neurological-expressed 1-like protein (Fragment) OS=Homo sapiens GN=HN1L PE=1 SV=1; Hematological and neurological-expressed 1-like protein (Fragment) OS=Homo sapiens GN=HN1L PE=1 SV=1; Hematological and neurological-expressed 1-like protein OS=Homo sapiens GN=HN1L PE=2 SV=2; Hematological and neurological expressed 1-like protein OS=Homo sapiens GN=HN1L PE=1 SV=1; Isoform 3 of Hematological and neurological expressed 1-like protein OS=Homo sapiens GN=HN1L; Isoform 2 of Hematological and neurological expressed 1-like protein OS=Homo sapiens GN=HN1L** | 2 | 3 | 0.08 | 1.20 | 0.023 |  | 2.00 | 3.00 | -0.25 | 1.20 | 0.008 |
| 109 | **Isoform 3 of Protein PRRC2C OS=Homo sapiens GN=PRRC2C** | 2 | 3 | 3.65 | -5.71 | 0.005 |  | 2.00 | 3.00 | 4.27 | -5.71 | 0.062 |
| 110 | **Lymphocyte-specific protein 1 OS=Homo sapiens GN=LSP1 PE=1 SV=1** | 2 | 3 | -0.50 | 0.72 | 0.354 |  | 2.00 | 3.00 | -1.22 | 0.72 | 0.024 |
| 111 | **Putative peptidyl-tRNA hydrolase PTRHD1 OS=Homo sapiens GN=PTRHD1 PE=1 SV=1** | 2 | 3 | 0.55 | 1.36 | 0.068 |  | 2.00 | 3.00 | 0.03 | 1.36 | 0.264 |

H=healthy; CRSwNP = CRS with nasal polyps; CRSsNP = CRS without nasal polyps
